# The omission of routine preoperative midazolam prescription is associated with increased preoperative sympathetic activation but not mortality: a propensity score matched, before-and-after study

**DOI:** 10.1186/s13741-025-00568-y

**Published:** 2025-07-30

**Authors:** Carolin Jung, Andre Gerdes, Hans-Joerg Gillmann, Thomas Stueber

**Affiliations:** 1 Department of Anesthesiology and Intensive Care Medicine, Hannover Medical School, Hannover, 30625 Germany; 2 Department of Anesthesiology and Intensive Care Medicine, Peine, 31226 Germany

**Keywords:** Preoperative anxiolysis, Midazolam, Preoperative blood pressure, Premedication, Propensity score matching

## Abstract

**Background:**

Guidelines recommend avoiding preoperative anxiolytic medication with midazolam. However, the risk–benefit ratio of preoperative midazolam prescriptions remains unclear. This study aimed to investigate the association between preoperative midazolam prescription and perioperative in-house mortality as well as preoperative cardiovascular stress.

**Methods:**

We performed a retrospective single-center propensity score-matched study in a university hospital in Germany before and after de-implementation of routine oral preoperative midazolam prescription in December 2018. We included adult patients who underwent general anesthesia between December 1, 2017, and November 31, 2019. Patients who received midazolam premedication before de-implementation were compared to those who did not receive midazolam after de-implementation. After propensity score matching, we estimated the treatment effects using regression modeling. The primary endpoint was inhospital mortality after general surgery. Secondary endpoints included pre-induction vital signs, duration of stay in the postanesthesia care unit, and medications administered.

**Results:**

After propensity score matching, we analyzed 7421 patients in each group. In this adjusted analysis, premedication with midazolam was not associated with mortality (*OR* 0.91, 95% *CI* 0.60 to 1.38, *p* = 0.662). Midazolam premedication was associated with significantly lower pre-induction blood pressures, with an estimated average treatment effect for systolic blood pressure of − 5.33 mmHg (*SE* 0.41, 95% *CI* − 6.13 to − 4.52 mmHg).

**Conclusions:**

Midazolam prescription was not associated with increased mortality in a large cohort of surgical patients but with a lower pre-induction blood pressure and heart rate, suggesting a potential reduction in cardiovascular stress.

**Supplementary Information:**

The online version contains supplementary material available at 10.1186/s13741-025-00568-y.

## Introduction

Routine prescription of anxiolytic premedication before surgery has long been a standard practice in hospitals worldwide. In addition to higher patient satisfaction, drug-induced anxiolysis is believed to improve hemodynamic stability while minimizing cardiovascular complications through effective stress shielding. Potential advantages of drug-induced anxiolysis are counterbalanced by concerns regarding adverse effects such as oversedation, risk of upper airway obstruction, extended wake-up durations following general anesthesia (Maurice-Szamburski et al. 2015), and risk of postoperative delirium. Current evidence concerning the risk–benefit ratio of medical anxiolysis using short-acting benzodiazepines is primarily derived from small observational studies and is still insufficient. Mounting evidence suggests that preoperative midazolam administration is not associated with an increased risk of postoperative delirium (Wang et al. 2021), and avoidance of anxiolytic premedication may be associated with increased mortality in older patients (Kowark et al. 2022). Nevertheless, various guidelines recommend against the routine use of drug-induced preoperative anxiolysis, irrespective of inadequate evidence regarding the risk–benefit ratio (Aldecoa et al. 2017; American Geriatrics Society Expert Panel on Postoperative Delirium in Older A 2015). In accordance with these guideline recommendations, clinical practice at the Hannover Medical School changed in 2018. On December 1, 2018, a new standard operating procedure discouraged the routine use of preoperative anxiolytic premedication. Anxiolytic premedication was since only to be prescribed in explicitly anxious patients. This study aimed to assess the effect of discontinuing routine preoperative midazolam prescriptions following a hospital-wide de-implementation directive on postoperative in-house mortality, hemodynamic changes before surgery, and further side effects, such as effects on preoperative peripheral oxygen saturation and the duration of postoperative anesthesia care unit (PACU) stay, in a large cohort of patients.

## Material and methods

### Study design and population

The Hannover Medical School Ethics Committee, Hannover, Germany (Chairperson Prof. B Schmidt), approved this retrospective study (Local Ethical Committee No. 10375_BO_SK_2022) on November 8, 2022, with a waiver of informed consent. This study was conducted in accordance with the Strengthening the Reporting of Observational Studies in Epidemiology (STROBE) guidelines (von Elm et al. 2014). Data analysis and statistical plans were written, date-stamped (permanently dated electronic signature), and recorded in the investigators’ files before the data were accessed. This study included all adult patients who received general anesthesia during surgical procedures at Hannover Medical School between December 1, 2017, and November 31, 2019. Hannover Medical School functions as a tertiary university hospital. All anesthesia procedures were digitally recorded. On December 1, 2018, the Department of Anaesthesia and Intensive Care Medicine updated the standard operating procedure (SOP) concerning premedication before surgery. The routine administration of midazolam was discouraged. The remaining indications for the prescription of preoperative midazolam in adults included anxiety, explicit requests from the patient, and prior awareness during previous surgical procedures. Anxiolytic premedication with midazolam was administered orally.

### Inclusion and exclusion criteria

**Fig. 1:**
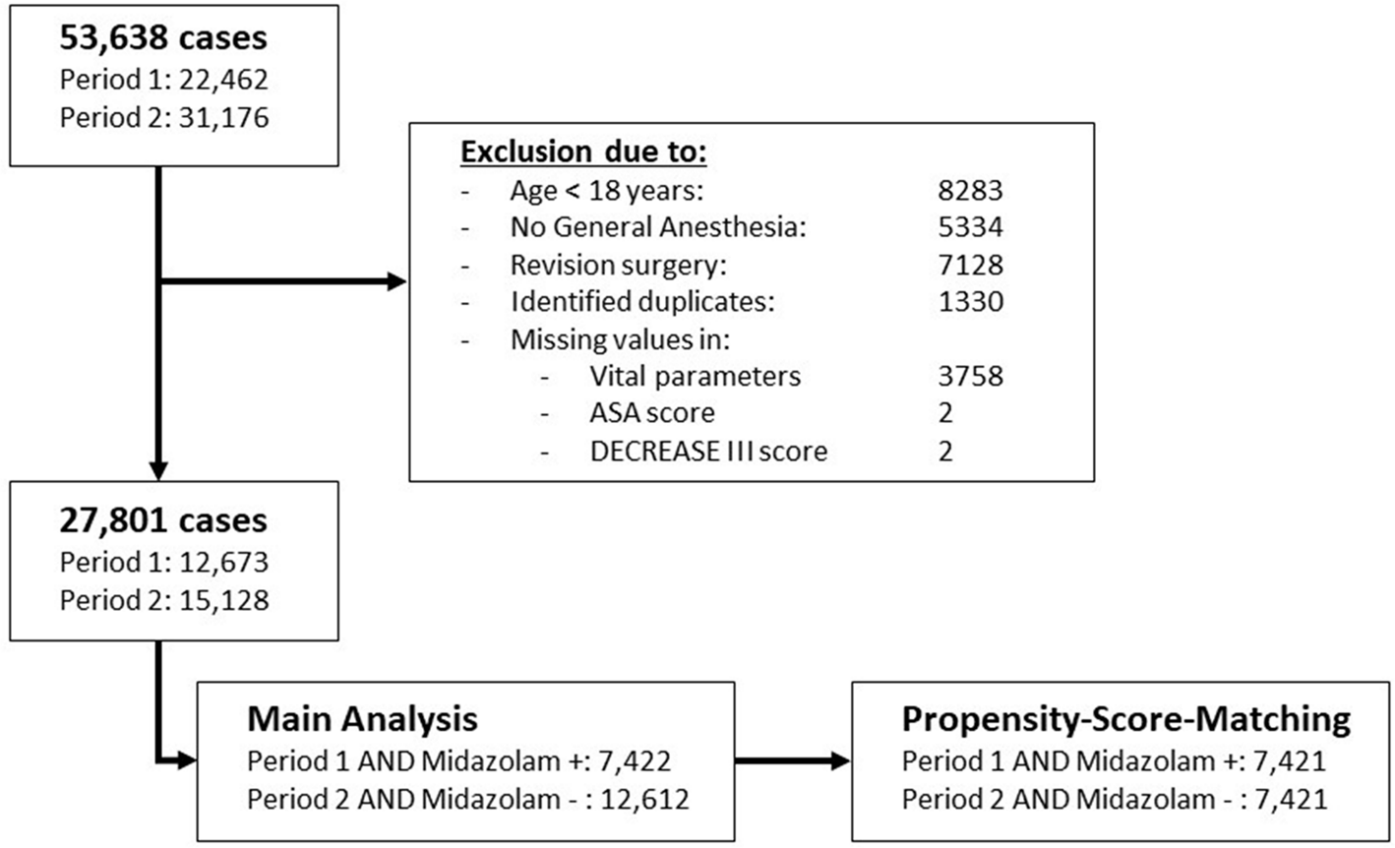
Consolidated Standards of Reporting Trials (CONSORT) diagram

The electronic anesthesia records of all adult patients who underwent surgery under general anesthesia in the year prior to and the year following the publication date of the SOP were incorporated. All records from December 1, 2017, to November 31, 2019, were collected. We excluded patients who (1) were younger than 18 years; (2) received repeated anesthesia for revision surgery; (3) had a surgical or medical procedure without general anesthesia, including those who received regional anesthesia only, sedation, or monitored care; or (4) had missing values in vital parameters, ASA, or the DECREASE III score (Fig. 1).

### Data collection and preprocessing

Perioperative data for all included patients were obtained from a digital anesthesia documentation data storage system (ANDOKlive, DATAPEC GmbH, Pliezhausen, Germany). A detailed description of data collection and data preprocessing is provided in the supplement.

### Study endpoints and main outcome measures

The primary endpoint was postoperative inhospital mortality. Secondary endpoints included preoperative blood pressure, heart rate, and Sp_O2_ measurements, as well as length of hospital stay, time to extubation, duration of PACU stay, and evaluation of pain in the postanesthesia care unit.

### Statistical analysis

We designed this study to have a power of 80%, with an *α* of 5%, to detect a between-group relative risk of 0.7 (Kowark et al. 2022) for the primary endpoint in adult patients with or without midazolam for premedication prior to induction of general anesthesia. We considered the missing values as not missing at random, which led us to forego imputation. Only cases with complete vital parameters and ASA and DECREASE III scores were included in the analysis. The DECREASE III score (Table S7) is a cardiovascular risk prediction score based on the Erasmus Risk Prediction model (Vidakovic et al. 2011) that incorporates surgery-related risk as well as patient-centered risk factors. This score was automatically calculated for all the electronic premedication records in our hospital. However, the validation of this risk score has never been published. Table S1 shows the number of missing values for each variable. Data management was conducted using IBM SPSS Statistics (Version 29.0.1.0 Armonk, NY, IBM Corp.). Statistical analysis was performed using R statistical software (R Foundation for Statistical Computing, Vienna, Austria, Version 4.4.1). Age, ASA classification, DECREASE III score, sex, length of surgery, and surgical specialty were all seen as confounding factors in the association between mortality and midazolam prescription. Consequently, they were included as covariates in the propensity score matching. To limit confounding by indication, the main analysis only involved group comparisons between patients who were prescribed midazolam during the liberal period (December 1, 2017, to November 31, 2018) and those who were not prescribed midazolam during the restrictive period (December 1, 2018, to November 31, 2019). Propensity score matching was used to estimate the effect of midazolam prescription on the primary and secondary outcome parameters using the *MatchIt* package (Ho et al. 2011). Propensity scores were calculated using 1:1 nearest neighbor matching with a caliper of 0.2 and without replacement. Balance between the treatment groups was evaluated using standardized mean differences. A standardized difference of ≤ 10% was considered adequate. Outcomes were estimated using generalized linear models with the *glm2* package (Marschner et al. 2011). Cluster-robust standard errors were calculated using the *marginaleffects* package (Arel-Bundock 2024), with the midazolam and non-midazolam groups serving as clusters. For the primary outcome, we used two-tailed testing with *α* set to 0.05 to determine statistical significance. We stated the estimated effects on secondary endpoints with confidence intervals, but without testing for significance to avoid multiple testing. To evaluate the minimum strength that an unmeasured confounder would require to produce a bias equal to the observed treatment-outcome association, we performed a post hoc sensitivity analysis calculating the *E*-value (VanderWeele et al. 2017) for the association of midazolam and the odds of a systolic blood pressure > 140 mmHg. Approximate *E*-values for odds ratios were calculated with a web-based calculator (Mathur et al. 2018).

### Post hoc* analyses*

Several post hoc exploratory analyses were performed. We explored a potential heterogeneous treatment effect of midazolam on the likelihood of systolic blood pressure exceeding 140 mmHg. This involved calculating the proportional difference and the associated confidence interval of patients with a systolic blood pressure greater than 140 mmHg, stratified by age group (18–64, 65–80, and > 80 years), sex, ASA score, type of surgery (cardiac and non-cardiac), and the use of combined general and regional anesthesia. In a further exploratory analysis, all patients who received a midazolam prescription were compared with all patients who did not receive a midazolam prescription, irrespective of the admission date (Tables S3 and S4).

## Results

Before propensity score matching, the standardized differences in covariates differed from − 0.219 to 0.307. After propensity score matching, all standardized differences in covariates ranged from − 0.008 to 0.014 (see supplement, Fig. 1B), indicating a good balance of baseline variables between the groups.

Hospital-wide orders for midazolam at the pharmacy decreased by almost 70% after the de-implementation of routine midazolam prescriptions. In 2018, a total of 10,060 doses of midazolam were ordered, whereas in 2019, the number dropped to 3220 doses. Of the 27,801 adult patients who underwent general anesthesia during both observation periods, 9938 were prescribed midazolam, whereas 17,638 did not receive midazolam. Patients without midazolam prescription were generally older and had higher ASA scores.

**Fig. 2:**
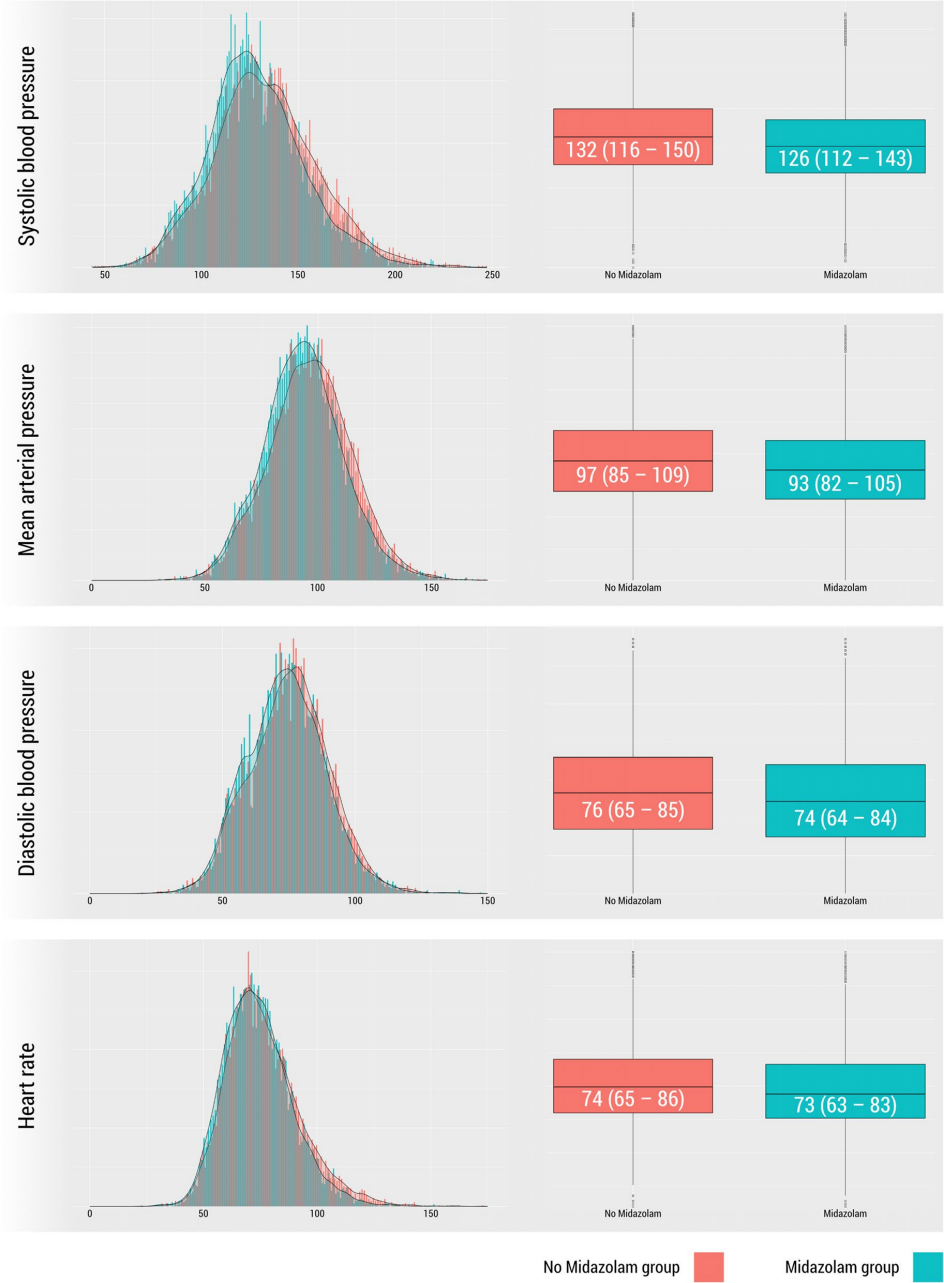
Histogram and density plot representing the distribution of systolic blood pressure, mean arterial pressure, diastolic blood pressure, and heart rate values in the cohort with midazolam in the liberal period (green) and without midazolam in the restrictive period (red)

During the first period, prior to the omission of routine preoperative midazolam prescriptions, 7422 patients received a midazolam prescription. In the second period, after the discouragement of midazolam prescription, 12,612 patients had no midazolam prescription. After propensity score matching, 7421 matched patients remained in each cohort. These patients were subsequently included in the main analysis. Baseline characteristics are presented in Table 1. The unadjusted analysis indicated lower inhospital mortality in the cohort of patients receiving midazolam during the first observation period compared to the cohort without midazolam prescriptions in period 2 (0.6% vs. 0.8%; *OR* 0.66, 95% *CI* 0.46 to 0.95). After adjustment with propensity score matching, the prescription of midazolam was associated with lower blood pressure and heart rate prior to anesthesia induction (Fig. 2), but not with inhospital mortality (Table 2). The odds of systolic blood pressure exceeding 140 mmHg or 170 mmHg prior to anesthesia induction were significantly higher in the cohort without midazolam prescription (Table 2). In the exploratory post hoc analysis, the attenuating effect of midazolam on the risk of a systolic blood pressure > 140 mmHg prior to anesthesia induction was consistent among all subgroups (see Table S5). The effect was most prominent in male patients, those aged 65–80 years, those classified as ASA II to III, and those undergoing cardiac surgery (Table S5). The peripheral oxygen saturation was only slightly lower in the midazolam cohort (Table 2). An Sp_O2_ of less than 92% occurred in 1.8% (*n* = 137) of patients receiving midazolam compared to 1.7% (*n* = 127) in the no-midazolam group (*OR* 1.08, 95% *CI* 0.85 to 1.38). The odds for a systolic blood pressure < 100 mmHg were higher in the midazolam cohort (11.4% (*n* = 843) vs. 9.8% (*n* = 725); *OR* 1.18, 95% *CI* 1.06 to 1.31). An exploratory post hoc analysis (Table S6) identified no differences in the risk of low systolic blood pressure between subgroups. There were no significant effects on time to extubation, duration of stay in the postanesthesia care unit, or length of hospital stay. Fewer antiemetic medications were administered in the midazolam group (Table 2). Midazolam was prescribed with a median dose of 7.5 mg orally (*IQR* [3.75 to 7.5 mg]).

**Table 1:** Baseline characteristics before and after propensity score matching

	**Unadjusted**		**Adjusted**	
	**Midazolam**	**No midazolam**	**Midazolam**	**No midazolam**
**Cases**	*N* = 7422	*N* = 12,612	*N* = 7421	*N* = 7421
**Age** (years) • **18 to 64** • **65 to 80** • > **80**	53 [57 to 65] 5450 (73%) 1734 (23%) 238 (3%)	57 [40 to 70] 8074 (64%) 3595 (29%) 943 (7%)	53 [37 to 65] 5449 (73%) 1734 (23%) 238 (3%)	53 [36 to 66] 5369 (72%) 1739 (23%) 313 (4%)
**Female sex**	4006 (54%)	6479 (51%)	4006 (54%)	4037 (54%)
**DECREASE III score**	31 [15 to 46]	30 [15 to 46]	31 [15 to 46]	31 [15 to 46]
**ASA score** • **1** • **2** • **3** • **4** • **5**	1225 (17%) 4145 (56%) 1945 (26%) 107 (1%) 0 (< 0.1%)	1621 (13%) 6590 (52%) 3948 (31%) 442 (4%) 11 (< 0.1%)	1224 (16%) 4145 (56%) 1945 (26%) 107 (1%) 0 ( < 0.1%)	1260 (17%) 4130 (56%) 1876 (25%) 152 (2%) 3 (< 0.1)
**Cardiac surgery**	456 (6%)	722 (6%)	456 (6%)	463 (6%)
**Combined general and regional anesthesia**	558 (8%)	858 (7%)	558 (8%)	524 (7%)
**Duration of surgery** (minutes)	80 [41 to 147]	70 [35 to 135]	80 [41 to 147]	76 [37 to 144]

Data are presented as number (proportion) or median [IQR]. *ASA* American Society of Anesthesiologists

**Table 2:** Outcome before and after propensity score matching

	**Unadjusted**		**Adjusted**		
	**Midazolam**	**No midazolam**	**Midazolam**	**No midazolam**	**Effect estimates**
**Cases**	*N* = 7422	*N* = 12,612	*N* = 7421	*N* = 7421	
**Inhospital mortality**	41 (0.6%)	105 (0.8%)	41 (0.6%)	50 (0.7%)	*OR* 0.91 (95% *CI* 0.60 to 1.38), *p* = 0.662
**Hospital length of stay** (days)	5 [3 to 9]	5 [3 to 9]	5 [3 to 9]	5 [3 to 9]	0.05 (SE 0.11, 95% CI −0.17 to 0.26)
**Vital parameters** - **Systolic arterial pressure** • ≥ **140 mmHg** • ≥ **170 mmHg** - **Mean arterial pressure** • **Diastolic arterial pressure** - **Heart rate** - **SpO**_**2**_	126 [112 to 143] 2218 (30%) 459 (6%) 93 [82 to 105] 74 [64 to 84] 73 [63 to 83] 99 [97 to 100]	135 [117 to 153] 5403 (43%) 1487 (12%) 98 [86 to 110] 76 [66 to 86] 74 [65 to 85] 99 [97 to 100]	126 [112 to 143] 2218 (30%) 459 (6%) 93 [82 to 105] 74 [64 to 84] 73 [63 to 83] 99 [97 to 100]	132 [116 to 150] 2892 (39%) 720 (10%) 97 [85 to 109] 76 [65 to 85] 74 [65 to 86] 99 [97 to 100]	− 5.33 (*SE* 0.41, 95% *CI* − 6.13 to − 4.52) *OR* 0.67 (95% *CI* 0.62 to 0.71) *OR* 0.62 (95% *CI* 0.55 to 0.70) − 3.14 (*SE* 0.29, 95% *CI* − 3.71 to − 2.57) − 1.61 (*SE* 0.24, 95% *CI* − 2.08 to − 1.14) − 2.5 (*SE* 0.25, 95% *CI* − 2.99 to − 2) − 0.30 (*SE* 0.04, 95% *CI* − 0.38 to − 0.23)
**Time to extubation** (minutes)	10 [6 to 16]	9 [5 to 15]	10 [6 to 16]	9 [5 to 15]	− 0.08 (*SE* 0.21, 95% *CI* − 0.34 to 0.50)
**Postoperative pain** (NPRS) • **Initial pain score** • **Maximal pain score**	2 [0 to 5] 4 [1 to 6]	2 [0 to 5] 3 [0 to 5]	2 [0 to 5] 4 [1 to 6]	2 [0 to 5] 4 [1 to 6]	0.05 (*SE* 0.09, 95% *CI* − 0.12 to 0.23) 0.06 (*SE* 0.09, 95% *CI* − 0.11 to 0.24)
**PACU medication** **• Piritramid (mg)** • **Clonidin** • **Granisetron** • **Dimenhydrinate**	8 [5 to 15] 832 (15%) 3278 (60%) 156 (3%)	6 [4 to 11] 1261 (14%) 5812 (64%) 224 (2%)	8 [5 to 15] 832 (15%) 3278 (60%) 156 (3%)	7 [5 to 12] 838 (16%) 3520 (65%) 151 (3%)	1.03 (*SE* 0.24, 95% *CI* 0.56 to 1.5) *OR* 0.93 (95% *CI* 0.79 to 1.1) *OR* 0.74 (95% *CI* 0.67 to 0.83) *OR* 1.06 (95% *CI* 0.64 to 1.74)
**Duration of PACU stay** (minutes)	70 [48 to 101]	65 [45 to 94]	70 [48 to 101]	66 [46 to 96]	− 0.37 (*SE* 2.92, 95% *CI* − 6.09 to 5.34)

*SpO*_*2*_ peripheral oxygen saturation, *NPRS* Numeric Pain Rating Scale, *PACU* postanesthesia care unit. Descriptive data are presented as median [IQR] or number (proportion). Blood pressures are given as mmHg. Data are presented as number (proportion) or median [IQR]. Effect estimates are given as the average treatment effect of the treated and cluster-robust standard error as well as the 95% confidence interval for continuous variables and as odds ratio with the 95% confidence interval for categorial variables

We also conducted an analysis comparing all patients who were prescribed midazolam with those who were not, irrespective of the period in which they were treated. This analysis revealed no relevant differences to the main analysis (Table S4). The *E*-value for the odds of having a systolic blood pressure exceeding 140 mmHg prior to anesthesia induction with a midazolam prescription was 1.74 for the effect estimate and 1.66 for the upper confidence interval.

## Discussion

In this propensity score-matched retrospective before-and-after study, oral premedication with midazolam before surgery was not associated with increased mortality or increased perioperative care times in the adjusted analysis. However, the range of the effect, as indicated by the confidence interval, encompasses both clinically important benefits and harm. Our study showed that midazolam prescription is associated with a significantly lower incidence of increased blood pressure and heart rate before anesthesia induction.

**Fig. 3:**
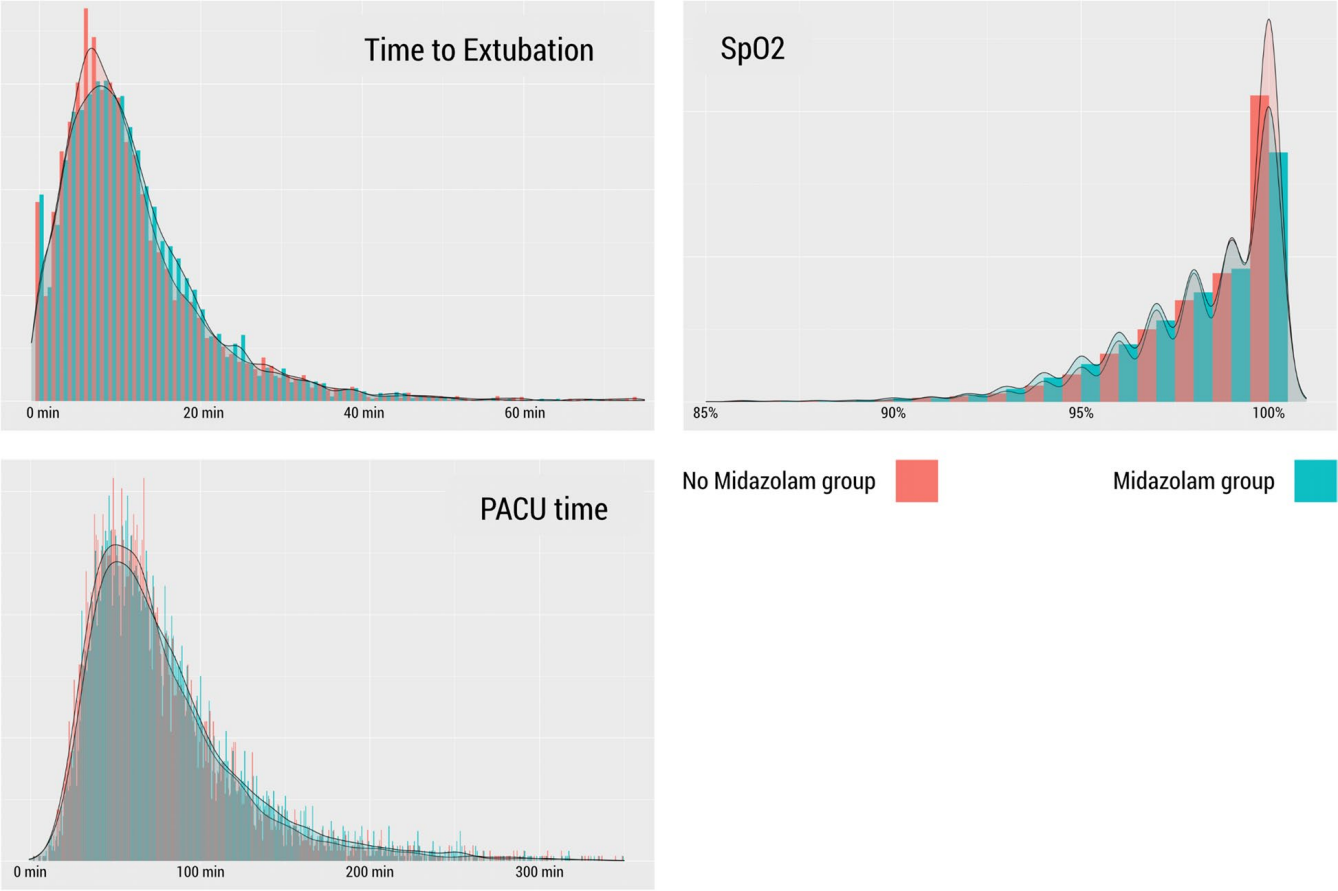
Histogram and density plot representing the distribution of the time to extubation, peripheral oxygen saturation (Sp_O2_), and duration of stay in the post-anesthesia care unit (PACU) in the cohort with midazolam in the liberal period (green) and without midazolam in the restrictive period (red)

The routine prescription of preoperative benzodiazepines has been largely abandoned because of concerns about benzodiazepine-induced postoperative delirium. However, large, randomized studies investigating the influence of premedication on postoperative outcomes are scarce. Lately, there has been mounting evidence from observational data that preoperative midazolam prescriptions may not be associated with an increased risk of delirium (Wang et al. 2021; Kowark et al. 2023; Stuff et al. 2022), and that perioperative acute inflammation may be a better predictor of postoperative cognitive changes than anesthetic medication (Aldecoa et al. 2024). A large prospective cohort of elderly patients aged ≥ 80 years (POSE cohort) (Kowark et al. 2022) indicated a reduced mortality hazard in patients receiving benzodiazepine premedication. In the relatively large randomized I-PROMOTE trial, postoperative complications did not differ between the midazolam and placebo groups (Kowark et al. 2023). Similar to our study, surgery without preoperative midazolam administration led to a higher preoperative blood pressure. There was no difference in 30-day mortality between the groups; however, this study was underpowered to detect mortality effects as only two events occurred. Furthermore, only a low dose of midazolam (3.75 mg) was investigated against placebo. The omission of routine midazolam prescriptions in our tertiary university hospital gave us the opportunity to assess the effect of this de-implementation strategy on mortality in a large cohort of patients. In our study, the prescription of a median dose of 7.5 mg orally administered midazolam was not associated with mortality or increased perioperative care time in a broad cohort of perioperative patients. Sp_O2_ was mildly lower in the midazolam group (Fig. 3). Given the equal distribution of patients with an Sp_O2_ of less than 92% between groups, this finding is unlikely to have any clinical relevance.

The hospital-wide strong reduction in ordered oral midazolam medication suggests that the intended de-implementation strategy indeed changed clinical practice. This clear effect on clinical practice was surprising, as there had already been a vivid debate about oral premedication even before the change in our SOP. The hospital-wide de-implementation likely decreased the risk of bias by indication as the standard operating procedure urged clinicians to avoid preoperative midazolam prescriptions. Thus, the decision to prescribe or withhold midazolam was no longer determined by clinicians’ preferences.

Our local standard operating procedure allows for the prescription of midazolam at the patient’s request. This could have introduced a bias by indication, as patients who were sicker or who underwent riskier surgery may have requested midazolam before surgery. This may have contributed to the significant association between the absence of midazolam prescriptions and mortality in our unadjusted analysis. We attempted to correct for these factors using propensity score-matched analysis. We included patients’ perioperative risk factors along with surgery-related risk factors in our adjusted model. However, the validation of this risk score has never been published. Patients who underwent revision surgery were excluded because of their complicated operative history, which could obscure the potential impact of perioperative midazolam administration on postoperative outcomes.

In accordance with the I-PROMOTE study, our study showed an association between midazolam prescription and lower blood pressures prior to anesthesia induction. The lower blood pressure and heart rate in our study suggests that anxiolytic premedication may have resulted in reduced sympathetic activation before surgery. This is in accordance with the findings of two small RCTs, in which premedication with low-dose midazolam had an attenuating effect on the immunological and endocrine stress responses during retrobulbar anesthesia in ophthalmologic patients (Kiefer et al. 1998; Heine et al. 2003). A large cohort study on the risk of preoperative blood pressure showed an independent association between high and low preoperative blood pressure and postoperative adverse events including mortality (Walco et al. 2024). The risk of adverse events increased in this study above a systolic blood pressure of 143 mmHg. In our study, the absence of a midazolam prescription was associated with a significantly higher incidence of patients with a preoperative blood pressure above 140 mmHg and 170 mmHg in a post hoc analysis of our data. Notably, the prescription of midazolam also increased the likelihood of preoperative hypotension, indicating potential harm for a subset of patients.

Our study also demonstrated an association between preoperative midazolam prescription and reduced application of antiemetic medication in the PACU. This suggests an antiemetic effect of midazolam, which has also been demonstrated in other studies (Grant et al. 2016). We believe that this finding strengthens the external validity of our analysis.

Our study is one of the largest to investigate the effects of preoperative midazolam prescription. We believe that the impact of perioperative midazolam on perioperative mortality, if present, would likely be minimal. However, because preoperative anxiolysis is a very frequent intervention, even small effects on adverse events would be relevant. Based on the currently available evidence, to which our study adds, an undifferentiated abandonment of preoperative anxiolytic medication for the general perioperative patient population is debatable. A post hoc analysis of our data further revealed some heterogeneous treatment effects regarding the attenuating effect of midazolam on the risk of having a systolic blood pressure > 140 mmHg (see Table S5). Pre-inductive hypertension was generally less frequent in patients aged 18–64, categorized as ASA I and undergoing noncardiac surgery. A potential attenuating effect on systolic blood pressure was observed in patients aged 80 years or younger and in patients with an ASA score of less than 4. While both sexes and both cardiac and noncardiac surgeries had lower pre-induction blood pressure in the midazolam group, the effect appeared to be more pronounced in male patients and in those undergoing cardiac surgery. Nevertheless, it must be noted that these potential heterogeneous treatment effects identified in this post hoc analysis are merely hypothesis generating and should be evaluated in prospective studies. Notably, while patients in the midazolam group also had an increased risk of having a systolic pressure < 100 mmHg prior to anesthesia induction, no vulnerable subgroups could be identified in the post hoc analysis of our data (Table S6). An increased risk of hypotension prior to anesthesia induction is an important safety concern that requires further investigation. Pre-induction hypotension is likely to worsen during anesthesia induction, thereby increasing the risk of perioperative organ dysfunction due to low tissue perfusion and increased vasopressor use. If future research can assess which specific populations benefit from or are harmed by anxiolytic premedication, this could pave the way for a personalized approach.

In addition to the large sample size, our study had several strengths. We extracted all data from electronic health records and preprocessed them transparently. Therefore, we believe that the risk of selection bias or outcome reporting bias is small. The hospital-wide omission of routine midazolam prescriptions gave us the opportunity to investigate our hypothesis and decrease the risk of bias by indication. However, it may be difficult to account for a treatment bias introduced by unmeasured or unmeasurable confounders. Patients who were prescribed midazolam may have appeared less frail or less sick with regard to cardiovascular disease irrespective of the clinical data we collected and balanced for. The fact that this study only included patients from a single center may limit its external validity. However, the demonstrated associations of preoperative midazolam prescription with antiemetic effects and blood pressure have also been shown in other observational studies, as well as in a randomized trial. Our study also has several limitations. Owing to the retrospective design of this study, we can only conclude an association between midazolam and our primary and secondary outcomes, but not causality. Although we tried to control for potential confounders with propensity score matching, we cannot rule out unmeasured residual confounding or confounding by the modeling strategy. The model we use for risk adjustment, as well as the DECREASE III score employed in this model, has not been subject to external validation, constituting a further limitation. The moderate *E*-values calculated for the association between pre-inductive hypertension and midazolam suggest a potential suggestibility of the observed effect to unmeasured confounding. Consequently, the robustness of this association should be interpreted with caution. Unfortunately, we lack data regarding preoperative blood pressures, postoperative delirium, and long-term and functional outcomes. As reducing midazolam prescriptions is primarily intended to mitigate the risk of delirium and postoperative cognitive dysfunction (POCD), this must be considered a significant limitation of our study. Since the prescription of midazolam had no effect on the length of the PACU or hospital stay, it is unlikely to have had a significant impact on major perioperative complications. However, no conclusions can be drawn about the incidence of delirium or POCD based on the length of the PACU or hospital stay. The evaluation of preoperative blood pressure is not part of the routine preoperative assessment in our institution. Therefore, preoperative blood pressure was not electronically recorded. Finally, we analyzed data concerning the prescription of preoperative midazolam, but we could not evaluate whether the premedication was actually administered. This may have been a source for exposure misclassification bias. At present, there is a paucity of data regarding the extent to which patients actually ingest midazolam following its prescription as a premedication agent prior to surgery. It is conceivable that patients exhibiting low levels of anxiety may be less inclined to administer their midazolam premedication. If the attenuating effect on perioperative cardiovascular stress is most likely to be observed in patients who are anxious before surgery, the magnitude of this bias on the outcomes mortality and cardiovascular stress would likely be minimal.

## Conclusions

Although the association between oral premedication with midazolam before surgery and mortality was not statistically significant in the adjusted analysis, the range of the confidence interval encompasses both clinically important benefits and harm. This leaves the result inconclusive. Whether there is a true effect on mortality, and whether specific subgroups may benefit from or be harmed by it, warrants prospective evaluation. Midazolam prescription was associated with a lower preoperative blood pressure and heart rate, indicating a reduction in cardiovascular stress.

## Supplementary Information

Additional file 1: Figure S1. Covariate balance displayed via mean standardized differences before (white) and after propensity score-matching of the main analysis. Figure S2. Distribution of Propensity Scores before and after propensity score-matching in the main analysis. Figure S3. Distribution of age between groups (by period) in the main analysis after propensity score-matching. Figure S4. Distribution of the DECREASE III score between groups (by period) in the main analysis after propensity score-matching. Figure S5. Distribution of Length of Surgery between groups (by period) in the main analysis after propensity score-matching. Figure S6. Distribution of the ASA score between groups (by period) in the main analysis after propensity score-matching. Figure S7. Distribution of the Sex between groups (by period) in the main analysis after propensity score-matching. Figure S8. Distribution of the proportion of patients with cardiac surgery between groups (by period) in the main analysis after propensity score-matching. Figure S9. Covariate balance displayed via mean standardized differences before (white) and after propensity score-matching in the analysis of all patients receiving midazolam vs. no midazolam, irrespective of the admission date. Figure S10. Distribution of Propensity Scores before and after propensity score-matching in the in the analysis of all patients receiving midazolam vs. no midazolam, irrespective of the admission date. Table S1. Missing values in the various subsets of this analysis. Table S2. Baseline characteristics of included patients in period 1 (December 1, 2017 – November 31, 2018) and 2 (December 1, 2018 – November 31, 2019). Table S3. Baseline characteristics before and after propensity-score matching of patients receiving Midazolam vs. no Midazolam during the observation period of two years irrespective of admission date. Table S4. Outcome before and after propensity-score matching of patients receiving Midazolam vs. no Midazolam during the observation period of two years irrespective of admission date. Table S5. Risk factors for systolic blood pressure > 140 mmHg in patients included in the propensity-score-matched main analysis. Table S6. Risk factors for systolic blood pressure. Table S7. DECREASE III risk score.

## Data Availability

No datasets were generated or analysed during the current study.

## Abbreviations

ASA: American Society of Anesthesiology
CI: Confidence interval
DAP: Diastolic arterial pressure
HR: Heart rate
IQR: Interquartile range
MAP: Mean arterial pressure
NPRS: Numeric Pain Rating Scale
PACU: Postanesthesia care unit
OR: Odds ratio
SBP: Systolic blood pressure
SE: Standard error
SOP: Standard operating procedure
SpO2: Peripheral oxygen saturation

## References

[CR1] Aldecoa C, Bettelli G, Bilotta F, Sanders RD, Audisio R, Borozdina A, et al. European Society of Anaesthesiology evidence-based and consensus-based guideline on postoperative delirium. Eur J Anaesthesiol. 2017;34(4):192–214. 28187050 10.1097/EJA.0000000000000594

[CR2] Aldecoa C, Bettelli G, Bilotta F, Sanders RD, Aceto P, Audisio R, et al. Update of the European Society of Anaesthesiology and Intensive Care Medicine evidence-based and consensus-based guideline on postoperative delirium in adult patients. Eur J Anaesthesiol. 2024;41(2):81–108. 37599617 10.1097/EJA.0000000000001876 PMC10763721

[CR3] American Geriatrics Society Expert Panel on Postoperative Delirium in Older A. American Geriatrics Society abstracted clinical practice guideline for postoperative delirium in older adults. J Am Geriatr Soc. 2015;63(1):142–50. 10.1111/jgs.13281 PMC5901697 25495432

[CR4] Arel-Bundock V, Greifer N, Heiss A. How to interpret statistical models using marginaleffects in R and Python. J Stat Softw. 2024;111(9):1–32. 10.18637/jss.v111.i09.

[CR5] Grant MC, Kim J, Page AJ, Hobson D, Wick E, Wu CL. The effect of intravenous midazolam on postoperative nausea and vomiting: a meta-analysis. Anesth Analg. 2016;122(3):656–63. 26332858 10.1213/ANE.0000000000000941

[CR6] Heine GH, Weindler J, Gabriel HH, Kindermann W, Ruprecht KW. Oral premedication with low dose midazolam modifies the immunological stress reaction after the setting of retrobulbar anaesthesia. Br J Ophthalmol. 2003;87(8):1020–4. 12881348 10.1136/bjo.87.8.1020 PMC1771790

[CR7] Ho D, Imai K, King G, Stuart E. MatchIt: nonparametric preprocessing for parametric causal inference. J Stat Softw. 2011;42(8):1–28.

[CR8] Kiefer RT, Weindler J, Ruprecht KW. The endocrine stress response after oral premedication with low-dose midazolam for intraocular surgery in retrobulbar anaesthesia. Eur J Ophthalmol. 1998;8(4):239–45. 9891896 10.1177/112067219800800407

[CR9] Kowark A, Berger M, Rossaint R, Schmid M, Coburn M, group PO-S. Association between benzodiazepine premedication and 30-day mortality rate: a propensity-score weighted analysis of the Peri-interventional Outcome Study in the Elderly (POSE). Eur J Anaesthesiol. 2022;39(3):210–8. 34817420 10.1097/EJA.0000000000001638 PMC8815825

[CR10] Kowark A, Keszei AP, Schneider G, Pilge S, Schneider F, Obert DP, et al. Preoperative midazolam and patient-centered outcomes of older patients: the I-PROMOTE randomized clinical trial. JAMA Surg. 2023. 10.1001/jamasurg.2023.6479 PMC10733850 38117527

[CR11] Marschner I. glm2: fitting generalized linear models with convergence problems. R J. 2011;3:12–5.

[CR12] Mathur MB, Ding P, Riddell CA, VanderWeele TJ. Web site and R package for computing E-values. Epidemiology. 2018;29(5):e45–7. 29912013 10.1097/EDE.0000000000000864 PMC6066405

[CR13] Maurice-Szamburski A, Auquier P, Viarre-Oreal V, Cuvillon P, Carles M, Ripart J, et al. Effect of sedative premedication on patient experience after general anesthesia: a randomized clinical trial. JAMA. 2015;313(9):916–25. 25734733 10.1001/jama.2015.1108

[CR14] Stuff K, Kainz E, Kahl U, Pinnschmidt H, Beck S, von Breunig F, et al. Effect of sedative premedication with oral midazolam on postanesthesia care unit delirium in older adults: a secondary analysis following an uncontrolled before-after design. Perioper Med (Lond). 2022;11(1):18. 35585564 10.1186/s13741-022-00253-4 PMC9118741

[CR15] VanderWeele TJ, Ding P. Sensitivity analysis in observational research: introducing the e-value. Ann Intern Med. 2017;167(4):268–74. 28693043 10.7326/M16-2607

[CR16] Vidakovic R, Poldermans D, Neskovic AN. Preoperative cardiac risk management. Acta Chir Iugosl. 2011;58(2):9–18. 10.2298/aci1102009v 21879645

[CR17] von Elm E, Altman DG, Egger M, Pocock SJ, Gotzsche PC, Vandenbroucke JP, et al. The Strengthening the Reporting of Observational Studies in Epidemiology (STROBE) statement: guidelines for reporting observational studies. Int J Surg. 2014;12(12):1495–9. 25046131 10.1016/j.ijsu.2014.07.013

[CR18] Walco JP, Rengel KF, McEvoy MD, Henson CP, Li G, Shotwell MS, et al. Association between preoperative blood pressures and postoperative adverse events. Anesthesiology. 2024;141(2):272–85. 38558232 10.1097/ALN.0000000000004991 PMC11233238

[CR19] Wang ML, Min J, Sands LP, Leung JM, the Perioperative Medicine Research G. Midazolam premedication immediately before surgery is not associated with early postoperative delirium. Anesth Analg. 2021;133(3):765–71. 33721875 10.1213/ANE.0000000000005482 PMC8373629

