# Supplementary material for: The omission of routine preoperative midazolam prescription is associated with increased preoperative sympathetic activation but not mortality: a propensity score matched, before-and-after study
Source: Perioper Med (Lond). 2025 Jul 30;14:81. doi: 10.1186/s13741-025-00568-y (PMC12308930; doi:10.1186/s13741-025-00568-y)

**Detailed description of data collection and preprocessing**

**Data collection**

Perioperative data for all included patients were obtained from a digital anaesthesia documentation data storage system (ANDOKlive, DATAPEC GmbH, Pliezhausen, Germany). ANDOKlive data encompassed preoperative, intraoperative, and postoperative information. Preoperative data included the American Society of Anaesthesiologists risk classification, DECREASE III risk score (Supplementary Table 6), age, and midazolam prescription. Perioperative data included time points, such as the initiation and end of anaesthesia and surgery, vital signs, ventilation metrics, intraoperative medications, and fluid administration. We extracted a maximum of five values documented prior to intubation for blood pressure, heart rate, and peripheral oxygen saturation (SpO2). The duration of surgery was defined as the time from incision to end of suturing. Time to extubation was defined as the time between the end of surgery and removal of the endotracheal tube. Postoperative data included the length of stay in the post-anaesthesia care unit (PACU), medications administered during the PACU stay, as well as the numeric pain rating scale recorded both on admission and during the PACU stay. In-house mortality data and data concerning the length of hospital stay were obtained from the Enterprise Clinical Research Data Warehouse (ECRDW) at Hannover Medical School (Hannover, Germany). Following the integration of perioperative anaesthesia data with data obtained via the ECRDW, the database was anonymized prior to the subsequent analysis.

**Data preprocessing**

The anonymised original database comprised 53,638 cases of documented anaesthesia during the investigation period, encompassing monitored anaesthesia care, regional anaesthesia, and general anaesthesia. Subsequently, we filtered the cases by age, excluding all patients younger than 18 years. All cases extracted twice according to the protocol number were excluded. Instances of multiple protocol numbers arose because of several invalid and incomplete premedication documents. Only the case in which the protocol number exhibited the highest number of data points was considered valid. Subsequently, we filtered the cohort to include only patients who underwent general anaesthesia. Finally, patients who underwent multiple anaesthetic procedures during the observation period were excluded from the study. A total of 27,801 patients were selected based on the date of anaesthesia. Period 1, characterized by routine midazolam use, included 12,673 cases, whereas period 2, during which midazolam was not routinely prescribed, included 15,128 cases.

For the analysis of vital data, we used the first available valid non-invasive blood pressure obtained prior to intubation. Systolic blood pressure values of > 300 mmHg or < 40 mmHg were considered invalid. The same accounted for mean arterial pressures above 179 mmHg or below 30 mmHg as well as diastolic blood pressures above 179 mmHg or below 20 mmHg. When no valid non-invasive blood pressure prior to intubation was available, the invasive blood pressure was utilized, provided valid data were recorded. The identical rules were applied to invasive blood pressure values. Furthermore, invasive blood pressure measurements were considered invalid if the systolic blood pressure was equal to the mean blood pressure and/or diastolic blood pressure (within ± 5 mmHg). With regard to the pre-induction heart rate, the first available valid heart rate was used for analysis. If the heart rate was < 30 or > 250 bpm, the value was assumed to be invalid, and the next available valid heart rate recorded prior to intubation was used. The in-hospital death data were extracted from the Clinical Data Warehouse. If no information regarding the patient’s death was recorded, we assumed that the patient did not die during the index hospital stay.

**Table S1**. Missing values in the various subsets of this analysis.

|  | Whole cohort | Study cohort | Main analysis - before PSM | Main analysis - propensity score matched | All patients receiving midazolam vs. no midazolam, irrespective of admission date |
| --- | --- | --- | --- | --- | --- |
| Cases | N = 31563 | N = 27801 | N = 20034 | N = 14844 | N = 19876 |
| Intrahospital death | 0 (0%) | 0 (0%) | 0 (0%) | 0 (0%) | 0 (0%) |
| Length of hospital stay | 2461 (7.8%) | 1982 (7.1%) | 1570 (7.8%) | 1265 (8.5%) | 1599 (8%) |
| Sex | 0 (0%) | 0 (0%) | 0 (0%) | 0 (0%) | 0 (0%) |
| Age | 0 (0%) | 0 (0%) | 0 (0%) | 0 (0%) | 0 (0%) |
| Cardiac surgery *y/n* | 0 (0%) | 0 (0%) | 0 (0%) | 0 (0%) | 0 (0%) |
| ASA classification | 2 (< 0.1%) | 0 (0%) | 0 (0%) | 0 (0%) | 0 (0%) |
| DECREASE III | 2 (< 0.1%) | 0 (0%) | 0 (0%) | 0 (0%) | 0 (0%) |
| Midazolam | 0 (0%) | 0 (0%) | 0 (0%) | 0 (0%) | 0 (0%) |
| Additional regional anesthesia | 0 (0%) | 0 (0%) | 0 (0%) | 0 (0%) | 0 (0%) |
| Peripheral oxygen saturation | 1148 (3.6%) | 0 (0%) | 0 (0%) | 0 (0%) | 0 (0%) |
| Heart rate | 1188 (3.8%) | 0 (0%) | 0 (0%) | 0 (0%) | 0 (0%) |
| Systolic blood pressure | 2973 (9.4%) | 0 (0%) | 0 (0%) | 0 (0%) | 0 (0%) |
| Mean arterial pressure | 3105 (9.8%) | 0 (0%) | 0 (0%) | 0 (0%) | 0 (0%) |
| Diastolic blood pressure | 2967 (9.4%) | 0 (0%) | 0 (0%) | 0 (0%) | 0 (0%) |
| Inital pain-score PACU (NPRS) | 15495 (49.1%) | 13228 (47.6%) | 9408 (47%) | 6878 (46.3%) | 9204 (46.3%) |
| Maximal pain-score PACU (NPRS) | 15518 (49.2%) | 13248 (47.7%) | 9421 (47%) | 6848 (46.1%) | 9218 (46.4%) |
| Duration of surgery | 0 (0%) | 0 (0%) | 0 (0%) | 0 (0%) | 0 (0%) |
| Time-to-extubation | 4782 (15.2%) | 3800 (13.7%) | 2785 (13.9%) | 2059 (13.9%) | 2635 (13.3%) |
| PACU time | 11825 (37.5%) | 9948 (35.8%) | 6977 (34.8%) | 5086 (34.3%) | 6838 (34.4%) |
| Granisetron | 9560 (30.3%) | 7920 (28.5%) | 5515 (27.5%) | 4010 (27%) | 5388 (27.1%) |
| Piritramid | 9560 (30.3%) | 7920 (28.5%) | 5515 (27.5%) | 4010 (27%) | 5388 (27.1%) |
| Dimenhydrinat | 9561 (30.3%) | 7921 (28.5%) | 5515 (27.5%) | 4010 (27%) | 5388 (27.1%) |
| Clonidin | 9560 (30.3%) | 7920 (28.5%) | 5515 (27.5%) | 4010 (27%) | 5388 (27.1%) |

Data are presented as number (proportion). Study cohort = remaining patients after exclusion of patients without complete vital parameters, DECREASE and ASA score. Main Analysis, includes only patients a) receiving midazolam in phase 1 or b) not receiving midazolam in phase 2. PSM = Propensity-score-matching; leads to exclusion of all patients who were unmatched during PSM. ASA = American Society of Anesthesiology. PACU = post-anesthesia care unit. NPRS =numeric pain rating scale.

**Table S2:** Baseline characteristics of included patients in period 1 (December 1, 2017 – November 31, 2018) and 2 (December 1, 2018 – November 31, 2019).

|  | Period 1 | Period 2 |  |
| --- | --- | --- | --- |
| Cases | N = 12673 | N = 15128 |  |
| Age (years)   - 18 to 64 - 65 to 80 - > 80 | 58 [43 to 71]  7898 (62%)  3825 (30%)  950 (8%) | 57 [40 to 70]  9717 (64%)  4285 (28%)  1126 (8%) |  |
| Female sex | 6378 (50%) | 7737 (51%) |  |
| DECREASE III score | 31 [18 to 46] | 30 [15 to 46] |  |
| ASA score   - 1 - 2 - 3 - 4 - 5 | 1471 (12%)  6510 (51%)  4347 (34%)  343 (3%)  2 (< 0.1%) | 1961 (13%)  7885 (52%)  4738 (31%)  531 (4%)  13 (< 0.1%) |  |
| Cardiac surgery | 725 (6%) | 851 (6%) |  |
| Combined general and regional anesthesia | 860 (7%) | 1010 (7%) |  |
| Duration of surgery (minutes) | 74 [38 to 144] | 70 [35 to 135] |  |
| Prescribed Midazolam   - Dose in mg | 7422 (59%)  7.5 [3.75 to 7.5] | 2516 (17%)  7.5 [7 to 7.5] |  |
| Vital parameters before anesthesia induction   - Systolic arterial pressure - Mean arterial pressure - Diastolic arterial pressure - Heart rate - Sp_O2_ | 131 [115 to 150]  96 [84 to 108]  75 [65 to 85]  72 [63 to 83]  99 [97 to 100] | 134 [117 to 164]  98 [86 to 110]  76 [66 to 86]  74 [64 to 85]  99 [97 to 100] |  |
| Intrahospital Death | 127 (1%) | 128 (0.8%) | OR 0.84  (95% CI 0.66 to 1.08) |
| Hospital length of stay (days) | 5 [3 to 9] | 5 [3 to 9] |  |

Data are presented as number (proportion) or median [IQR]. ASA, American Society of Anaesthesiologists.

**Table S3:** Baseline characteristics before and after propensity-score matching of patients receiving Midazolam vs. no Midazolam during the observation period of two years irrespective of admission date.

|  | Unadjusted |  | Adjusted |  |
| --- | --- | --- | --- | --- |
|  | **Midazolam** | **No Midazolam** | **Midazolam** | **No Midazolam** |
| Cases | N = 9938 | N = 17863 | N = 9937 | N = 9937 |
| Age in years   - 18 to 64 - 65 to 80 - > 80 | 53 {38 to 67]  7093 (71%)  2424 (24%)  421 (4%) | 60 (45-72 [18-102])  10522 (59%)  5686 (32%)  1655 (9.3%) | 53 (38-67 [18-95])  7092 (71%)  2424 (24%)  421 (4%) | 54 [37 to 67]  6972 (70%)  2442 (24%)  523 (5%) |
| Female sex | 5264 (53%) | 8851 (50%) | 5263 (53%) | 5312 (53%) |
| DECREASE III score | 31 [15 to 46] | 31 [16 to 47] | 31 [15 to 46] | 31 [15 to 46] |
| ASA score   - 1 - 2 - 3 - 4 - 5 | 1565 (16%)  5440 (55%)  2735 (28%)  196 (2%)  2 (< 0.1%) | 1867 (10%)  8955 (50%)  6350 (36%)  3678 (3.8%)  13 < 0.1%) | 1564 (16%)  5440 (55%)  2735 (28%)  196 (2%)  2 (< 0.1%) | 1559 (16%)  5447 (55%)  2737 (28%)  191 (2%)  3 (< 0.1%) |
| Cardiac surgery | 585 (6%) | 991 (5.5%) | 585 (6%) | 618 (6%) |
| Combined general and regional anesthesia | 710 (7%) | 1160 (6.5%) | 710 (7%) | 674 (7%) |
| Duration of surgery (minutes) | 77 [39 to 145] | 69 [35 to 135] | 77 [39 to 145] | 74 [37 to 144] |

Data are presented as number (proportion) or median [IQR]. ASA = American Society of Anaesthesiologists.

**Table S4:** Outcome before and after propensity-score matching of patients receiving Midazolam vs. no Midazolam during the observation period of two years irrespective of admission date.

|  | Unadjusted |  | Adjusted |  |  |
| --- | --- | --- | --- | --- | --- |
|  | **Midazolam** | **No Midazolam** | **Midazolam** | **No Midazolam** | **Effect estimates** |
| Cases | N = 9938 | N = 17863 | N = 9937 | N = 9937 |  |
| Death | 64 (0.6%) | 191 (1.1%) | 64 (0.6%) | 85 (0.9%) | OR 0.79 (95% CI 0.57 to 1.08),  p = .138 |
| Hospital length  of stay (days) | 5 [3 to 9] | 5 [3 to 9] | 5 [3 to 9] | 5 [3 to 9] | -0.15 (SE 0.1, 95% CI -0.36 to 0.46) |
| Vital parameters  - Systolic arterial pressure  - ≥ 140 mmHg  - ≥ 170 mmHg  - Mean arterial pressure  - Diastolic arterial pressure  - Heart rate  - SpO_2_ | 128 [113 to 146]  3285 (33%)  786 (8%)  94 [83 to 106]  74 [64 to 84]  73 [63 to 83]  99 [97 to 100] | 136 [118 to 156] 8059 (45%)  2402 (13%)  99 [86 to 111]  76 [66 to 86]  73 [64 to 84]  99 [97 to 100] | 128 [113 to 146]  3285 (33%)  786 (8%)  94 [83 to 106]  74 [64 to 84]  73 [63 to 83]  99 {97 to 100] | 133 [116 to 152]  4047 (41%)  1077 (11%)  97 [85 to 109]  76 [66 to 85]  74 (65 to 85]  99 [97 to 100] | -4.27 (SE 0.36, 95% CI -4.98 to -3.56)  OR 0.73 (95% CI 0.69 to 0.77)  OR 0.72 (95% CI 0.66 to 0.80)  -2.54 (SE 0.26, 95% CI -3.04 to -2.04)  -1.25 (SE 0.21, 95% CI -1.66 to -0.84)  -1.51 (SE 0.22, 95% CI -1.94 to -1.09)  -0.16 (SE 0.04, 95% CI -0.23 to -0.09) |
| Time to extubation, minutes | 10 [6 to 15] | 9 [5 to 15] | 10 [6 to 15] | 9 [5 to 15] | 0.04 (SE 0.19, 95% CI -0.34 to 0.41) |
| Postoperative pain (NPRS)  - Initial pain  Score  - Maximal pain  score | 2 [0 to 5]  4 [0 to 6] | 2 [0 to 5]  3 [0 to 5] | 2 [0 to 5]  4 [1 to 6] | 2 [0 to 5]  4 [1 to 6] | 0,01 (SE 0.07, 95% CI -0.14 to 0.15)  -0,02 (SE 0.07, 95% CI -0.16 to 0.12) |
| PACU medication  - Piritramid (mg)  - Clonidin  - Granisetron  - Dimenhydrinat | 7 [5 to 14]  1068 (15%)  4443 (61%)  195 (2.7%) | 6 [4 to 11]  1676 (13%)  8074 (64%)  310 (2.5%) | 7 (5 to 14]  1068 (15%)  4442 (61%)  195 (3%) | 7 [4 to 12]  1055 (15%)  4624 (64%)  203 (3%) | 0,71 (SE 0.18, 95% CI 0.35 to 1.07)  OR 1.01 (95% CI 0.88 to 1.16)  OR 0.87 (95% CI 0.80 to 0.96)  OR 0.92 (95% CI 0.62 to 1.37) |
| Duration of PACU stay (minutes) | 69 [48 to 100] | 65 [45 to 94] | 69 [48 to 100] | 66 [46 to 95] | 1.58 (SE 4.38, 95% CI -6.99 to 10.2) |

Data are presented as number (proportion) or median [IQR]. Blood pressures are given as mmHg. SBP, systolic blood pressure; MAP, mean arterial pressure; DBP, diastolic blood pressure; HR, heart rate; Sp_O2_, peripheral oxygen saturation; NPRS, numeric pain rating scale; PACU, post anesthesia care unit. Effect estimates are given as the Average treatment effect of the treated and cluster-robust standard error as well as the 95% confidence interval for continuous variables and as Odds Ratio with the 95% confidence interval for categorial variables.

**Table S5.** Risk factors for systolic blood pressure > 140 mmHg in patients included in the propensity-score-matched main analysis.

|  | No. of patients with systolic arterial pressure > 140 mmHg |  | Between-group difference  ( 95% CI), % |
| --- | --- | --- | --- |
|  | **Midazolam** | **No Midazolam** |  |
| Cases | 2218/7421 (30%) | 2892/7421 (39%) |  |
| Age   - 18 – 64 - 65-80 - > 80 | 1307/5449 (24%)  782/1734 (45.1%)  129/238 (54.2%) | 1697/5369 (31.6%)  1008/1739 (58%)  187/313 (59.7%) | - 7.6 (-10.8 to -4.4)  - 12.8 (-17.5 to -8.3)  -5.5 (-16.6 to 5.6) |
| Sex   - Female - Male | 1168/4006 (29.2%)  1050/3415 (30.7%) | 1472/4037 (36.5%)  1420/3384 (42%) | -7.3 (-10.8 to -3.7)  -11.3 (-15.1 to -7.5) |
| ASA score   - 1 - 2 - 3 - 4 - 5 | 230/1224 (18.8%)  1202/4145 (29%)  752/1945 (38.7%)  34/107 (31.8%)  0/0 (0%) | 328/1260 (26%)  1578/4130 (38.2%)  922/1876 (49.1%)  63/152 (41.4%)  1/3 (33.3%) | -7.2 (-14.1 to -0.3)  -9.2 (-12.7 to -5.7)  -10.4 (-15.1 to -5.7)  - 9.6 (-29.4 to 10.2) |
| Cardiac surgery  No cardiac surgery | 177/456 (38.8%)  2041/6965 (29.3%) | 245/463 (52.9%)  2647/6958 (38%) | -14.1 (-23.6 to -4.6)  -8.7 (-11.4 to -6) |
| Combined general and regional anesthesia | 148/558 (26.5%) | 197/524 (37.6%) | -11.1 (-20.9 to -1.3) |

Data are presented in (number/total number (proportion) and the proportional difference in percent with the respective 95% CI (in percent). ASA = American Society of Anaesthesiologists.

**Table S6.** Risk factors for systolic blood pressure < 100 mmHg in patients included in the propensity-score-matched main analysis.

|  | No. of patients with systolic arterial pressure < 100 mmHg |  | Between-group difference  ( 95% CI), % |
| --- | --- | --- | --- |
|  | **Midazolam** | **No Midazolam** |  |
| Cases | 843/7421 (11%) | 725/7421 (9,8%) |  |
| Age   - 18 – 64 - 65-80 - > 80 | 642/5449 (11.8%)  181/1734 (10.4%)  20/238 (8.4%) | 561/5369 (10.4%)  142/1739 (8.2%)  22/313 (7%) | 1.4 (-2.2 to 5)  2.2 (-4.1 to 8.5)  1.4 (-14.7 to 17.6) |
| Sex   - Female - Male | 493/4006 (12.3%)  350/3415 (10%) | 426/4037 (10.6%)  299/3384 (7.7%) | 1.7 (-2.4 to 5.8)  2.3 (-2.1 to 6.7) |
| ASA score   - 1 - 2 - 3 - 4 - 5 | 127/1224 (10.4%)  491/4145 (11.8%)  215/1945 (11.1%)  10/107 (9.3%)  0/0 (0%) | 113/1260 (9%)  410/4130 (10%)  174/1876 (9.3%)  27/152 (17.8%)  1/3 (33.3%) | 1.4 (-6.1 to 8.9)  1.8 (-2.3 to 5.9)  1.8 (-4.2 to 7.8)  -8.5 (-31.6 to 14.6) |
| Cardiac surgery  No cardiac surgery | 46/456 (10.1%)  797/6965 (11.4%) | 38/463 (8.2%)  687/6958(9.9%) | 1.9 (-10.4 to 14.2)  1.5 (-1.6 to 4.6) |
| Combined general and regional anesthesia | 60/558 (10.8%) | 49/524 (9.4%) | 1.4 (-9.9 to 12.7) |

Data are presented in (number/total number (proportion) and the proportional difference in percent with the respective 95% CI (in percent). ASA = American Society of Anaesthesiologists.

**Table S7.** DECREASE III risk score.

| **DECREASE III risk score** |
| --- |
| **Age**  **≤ 40 years 0 points**  **41 – 50 years 5 points**  **51 – 60 years 10 points**  **61 – 70 years 15 points**  **71 – 80 years 20 points**  **> 80 years 25 points** |
| **Sex**  **Female 0 points**  **Male 1 point** |
| **Type of surgery**  **Low risk 0 points**  (Mamma surgery, dental surgery, ophthalmic surgery, gynecological (without intraabdominal) surgery, plastic and reconstructive surgery, orthopedic or trauma surgery, and urogenital surgery)  **Medium risk 30 points**  (Intraabdominal surgery, intrathoracic surgery, head and neck surgery, pelvic or spine surgery, neurosurgery, transplant surgery, and endovascular interventions)  **High Risk** **35 points**  (Aortic surgery, thrombendarterectomy, and cardiac surgery) |
| **Clinical risk factors**  **Coronary artery disease** **2 points**  **Renal failure 2 points**  **Diabetes mellitus 5 points**  **Hypertension 5 points**  **Heart failure 10 points** |

A maximum score of 85 points can be achieved.

**Figure S1**: Covariate balance displayed via mean standardized differences before (white) and after propensity score-matching of the main analysis.


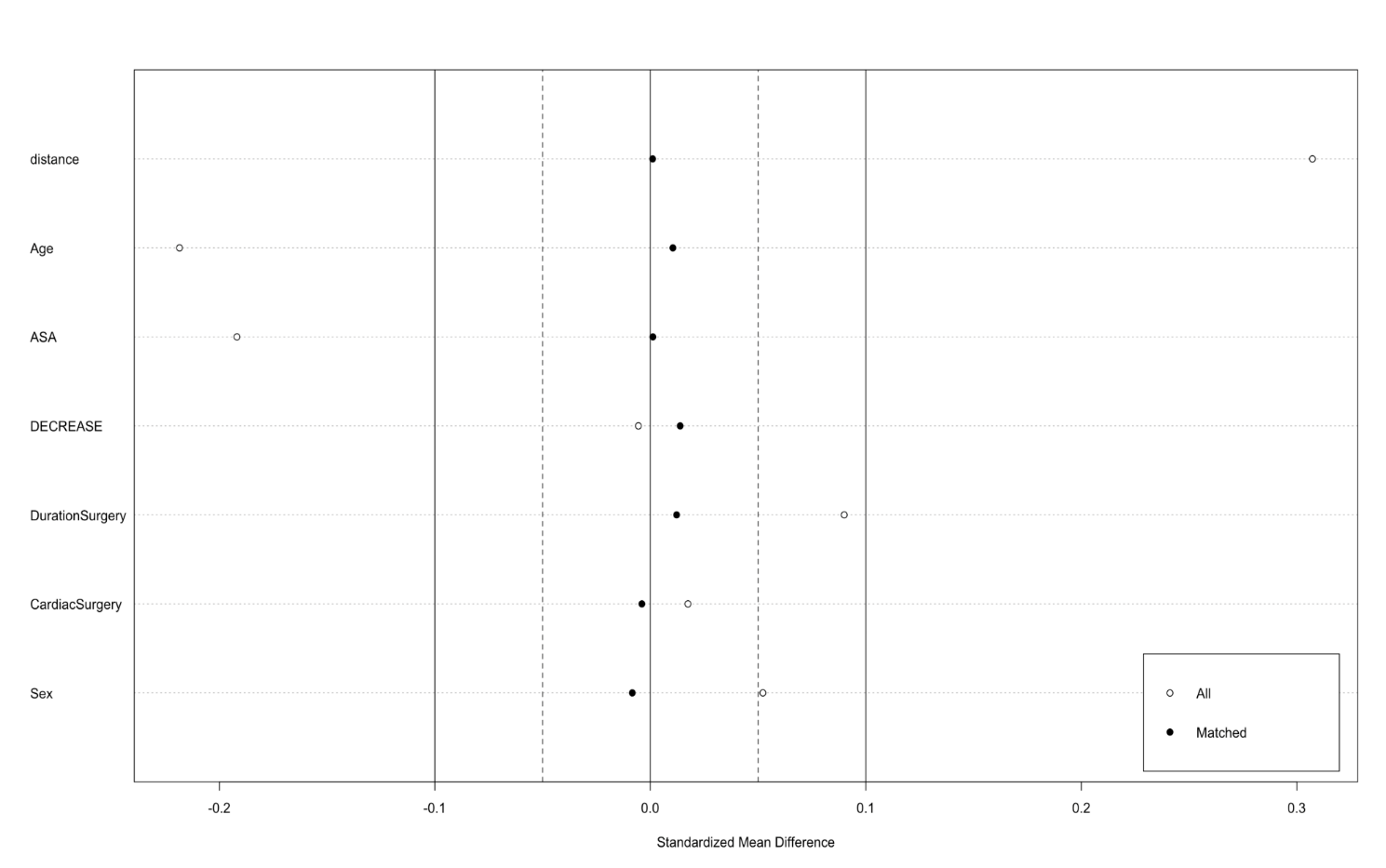


**Figure S2**: Distribution of Propensity Scores before and after propensity score-matching in the main analysis.


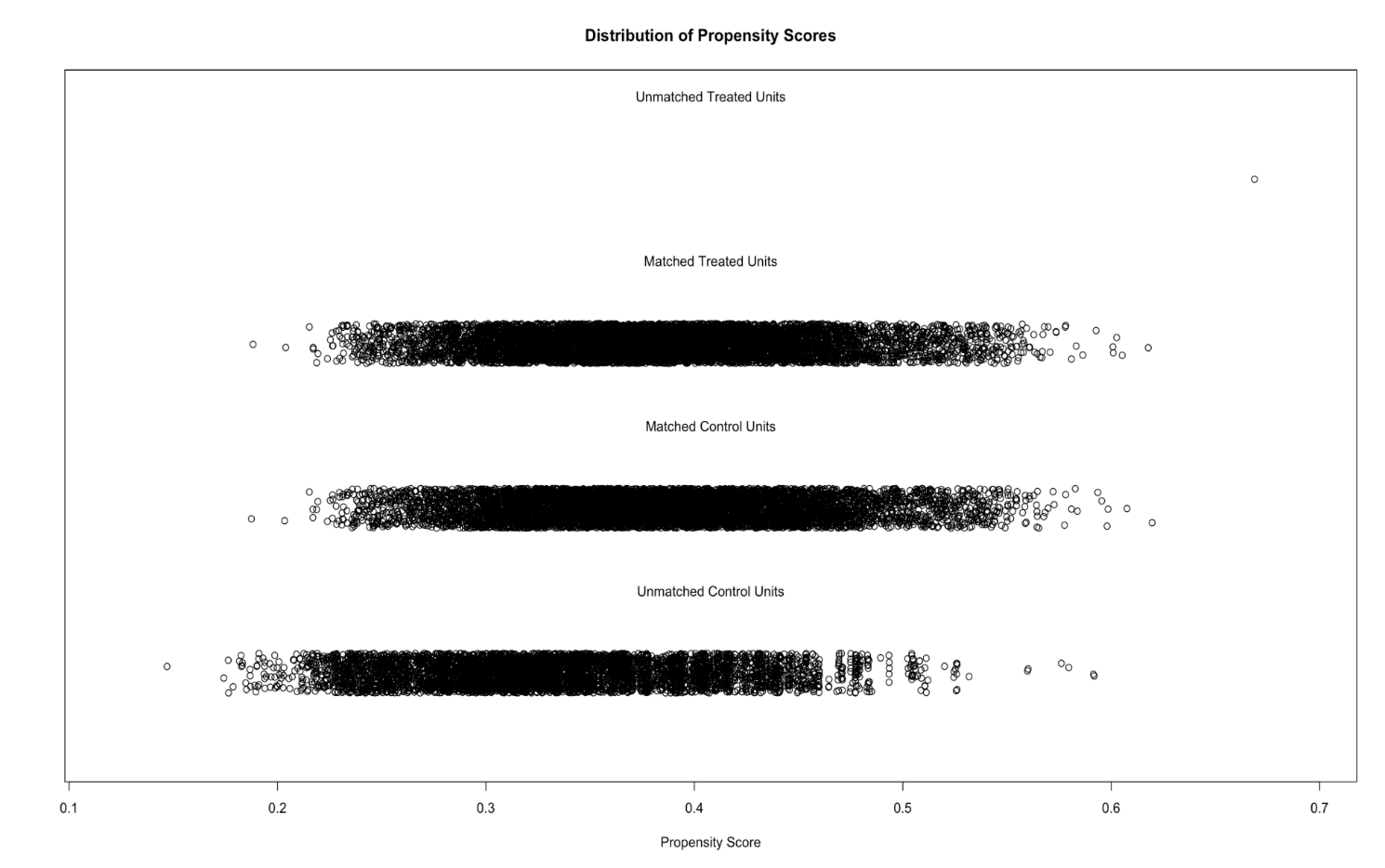


**Figure S3**: Distribution of age between groups (by period) in the main analysis after propensity score-matching.

**
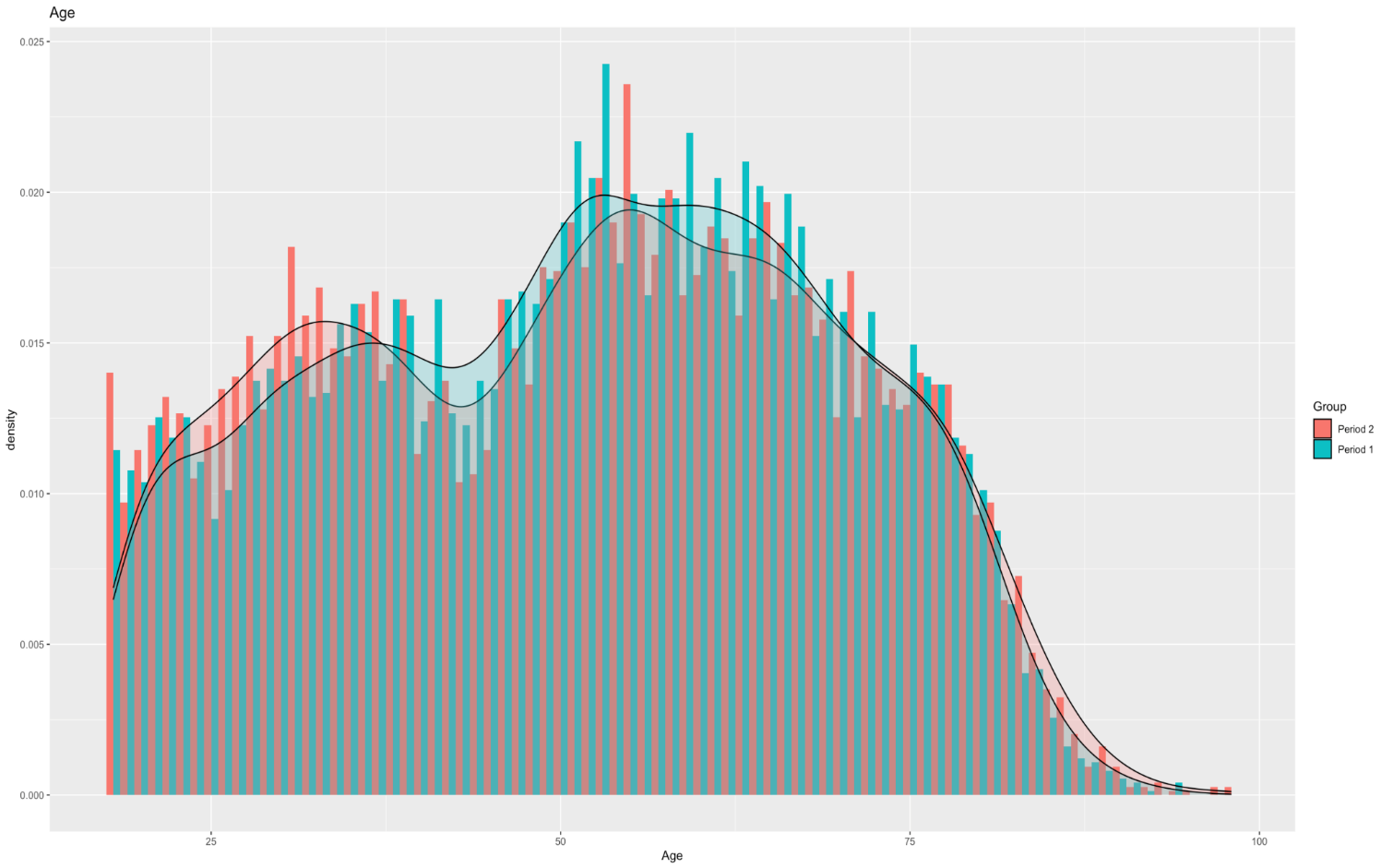
**

**Figure S4**: Distribution of the DECREASE III score between groups (by period) in the main analysis after propensity score-matching.


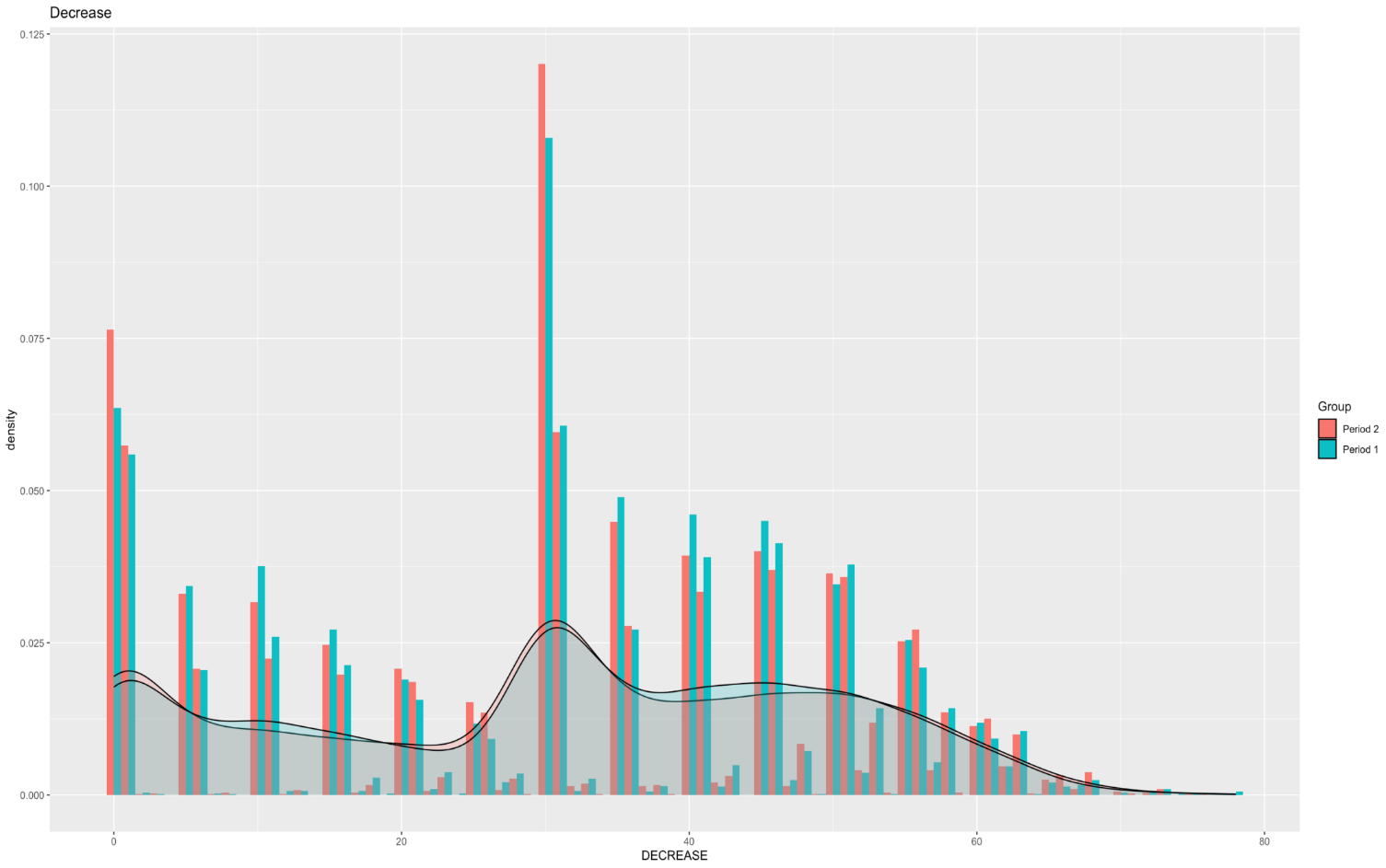


**Figure S5**: Distribution of Length of Surgery between groups (by period) in the main analysis after propensity score-matching.

**
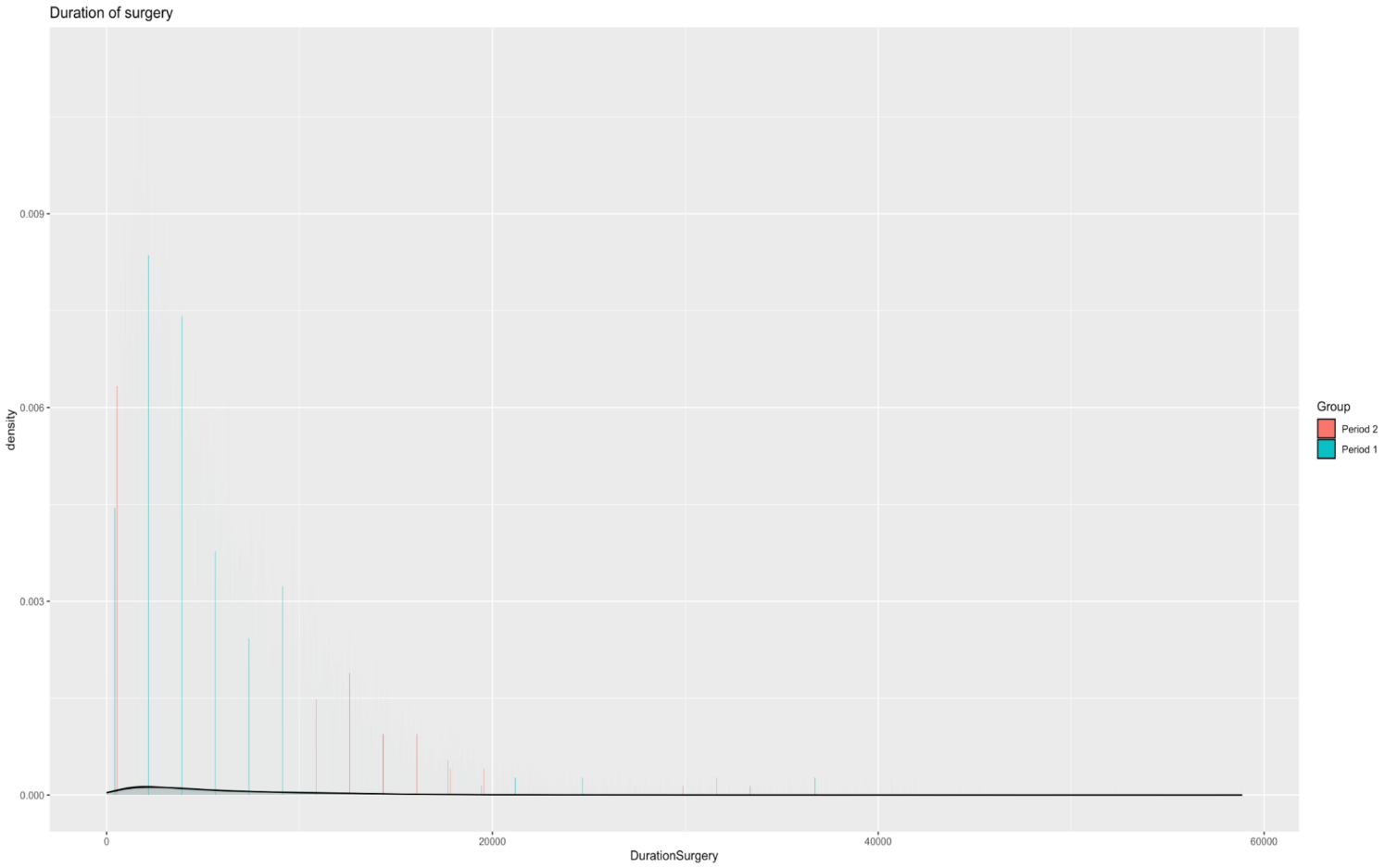
**

**Figure S6**: Distribution of the ASA score between groups (by period) in the main analysis after propensity score-matching.

**
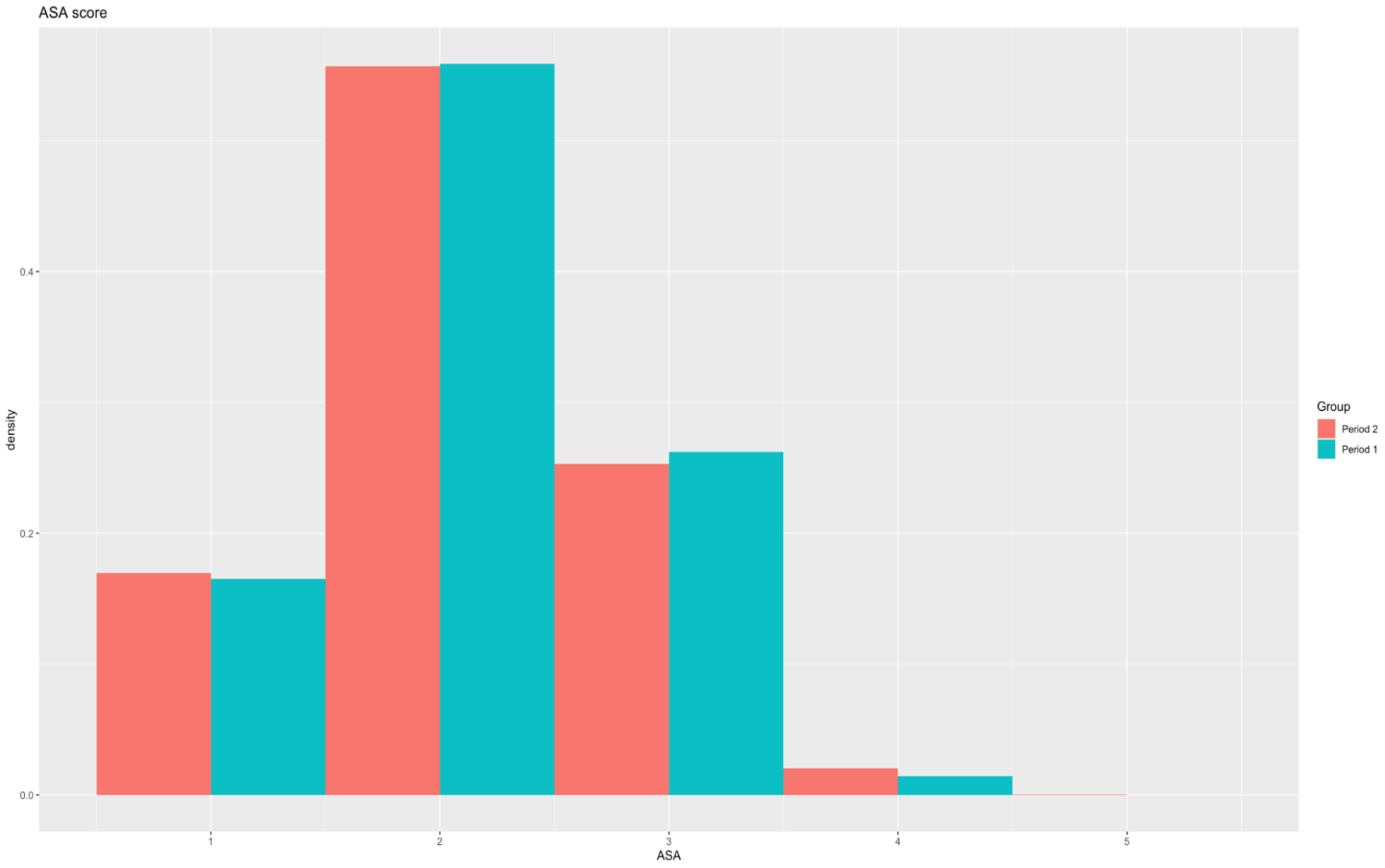
**

**Figure S7**: Distribution of the Sex between groups (by period) in the main analysis after propensity score-matching.


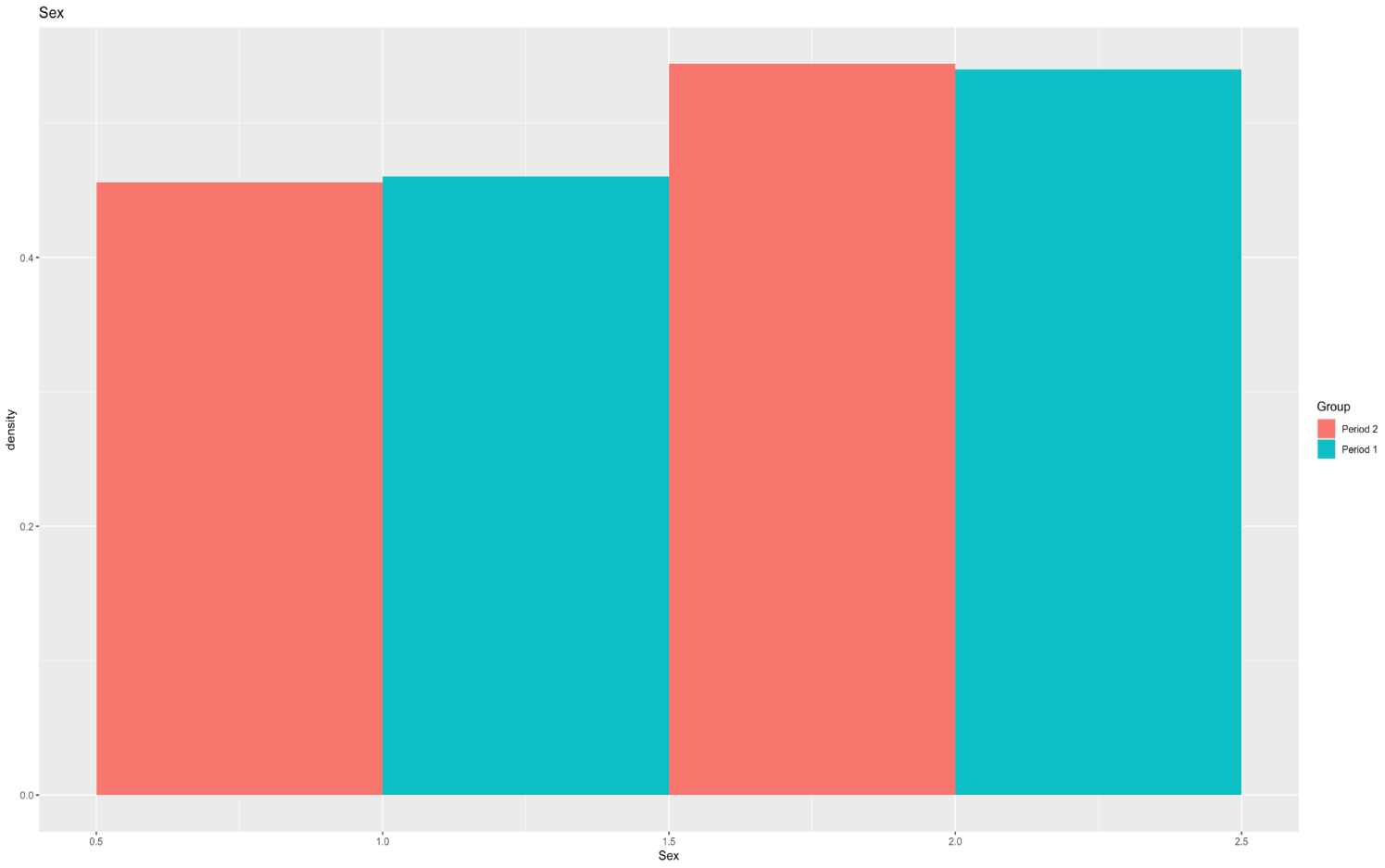


**Figure S8**: Distribution of the proportion of patients with cardiac surgery between groups (by period) in the main analysis after propensity score-matching.


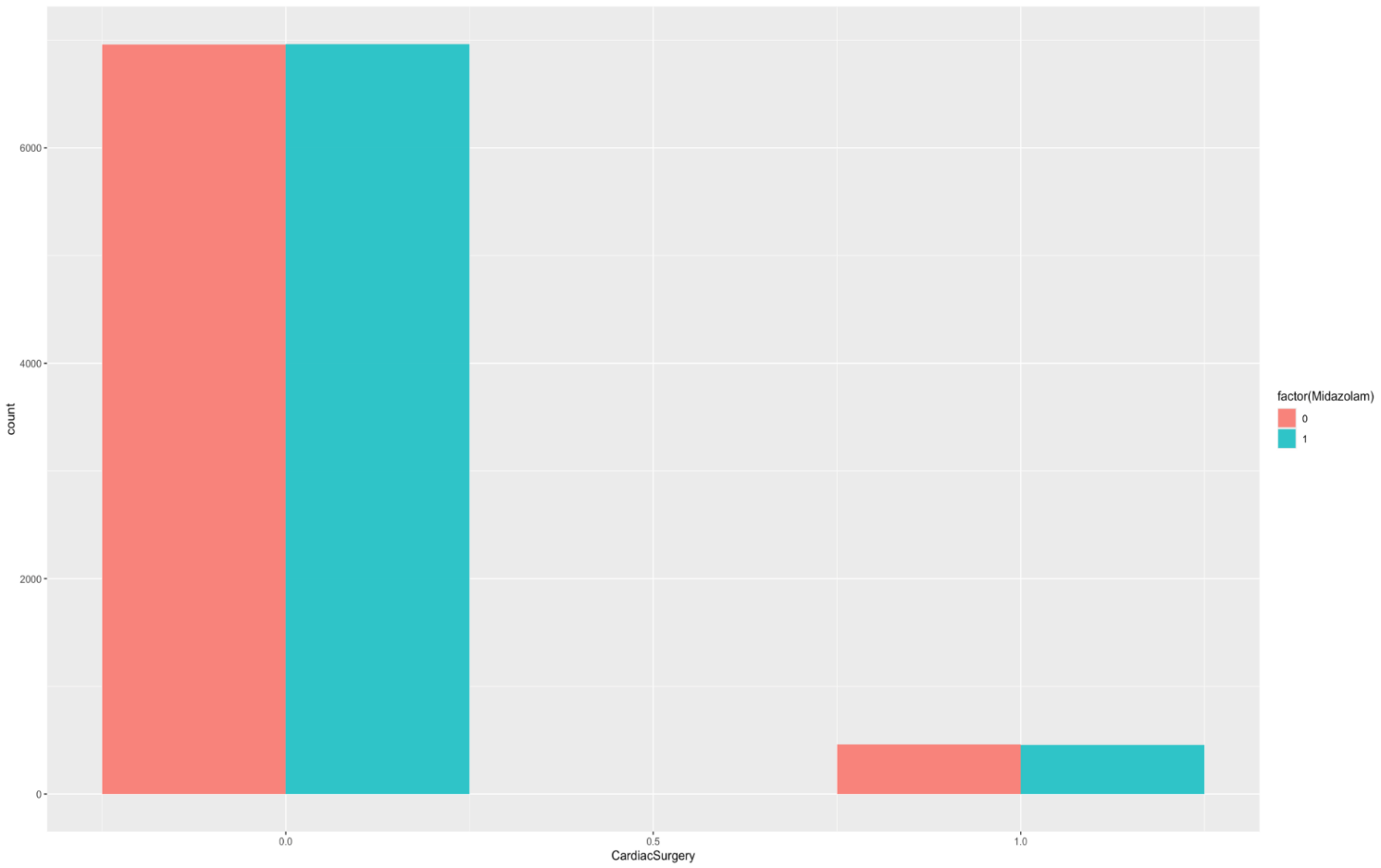


**Figure S9**: Covariate balance displayed via mean standardized differences before (white) and after propensity score-matching in the analysis of all patients receiving midazolam vs. no midazolam, irrespective of the admission date.


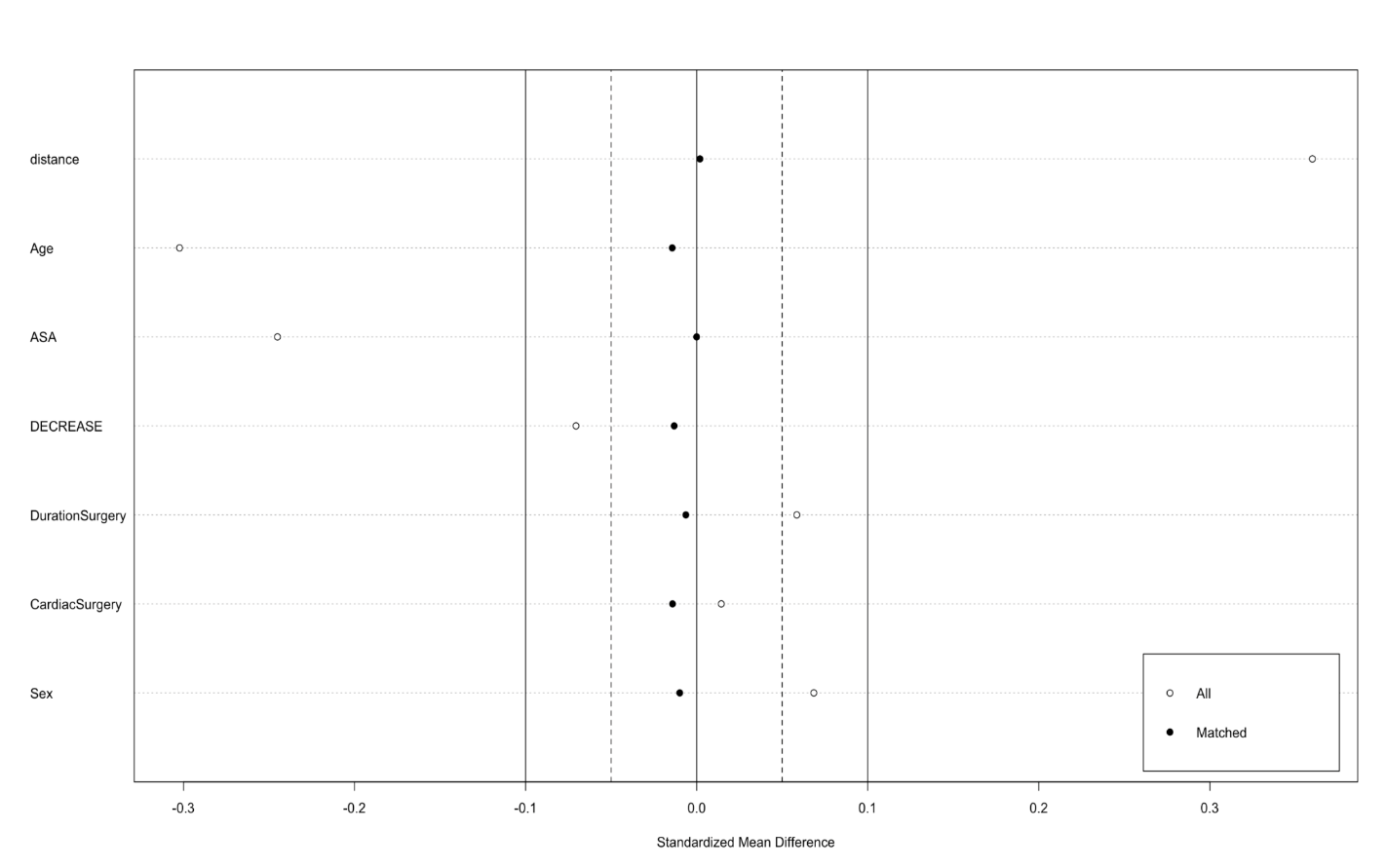


**Figure S10**: Distribution of Propensity Scores before and after propensity score-matching in the in the analysis of all patients receiving midazolam vs. no midazolam, irrespective of the admission date.


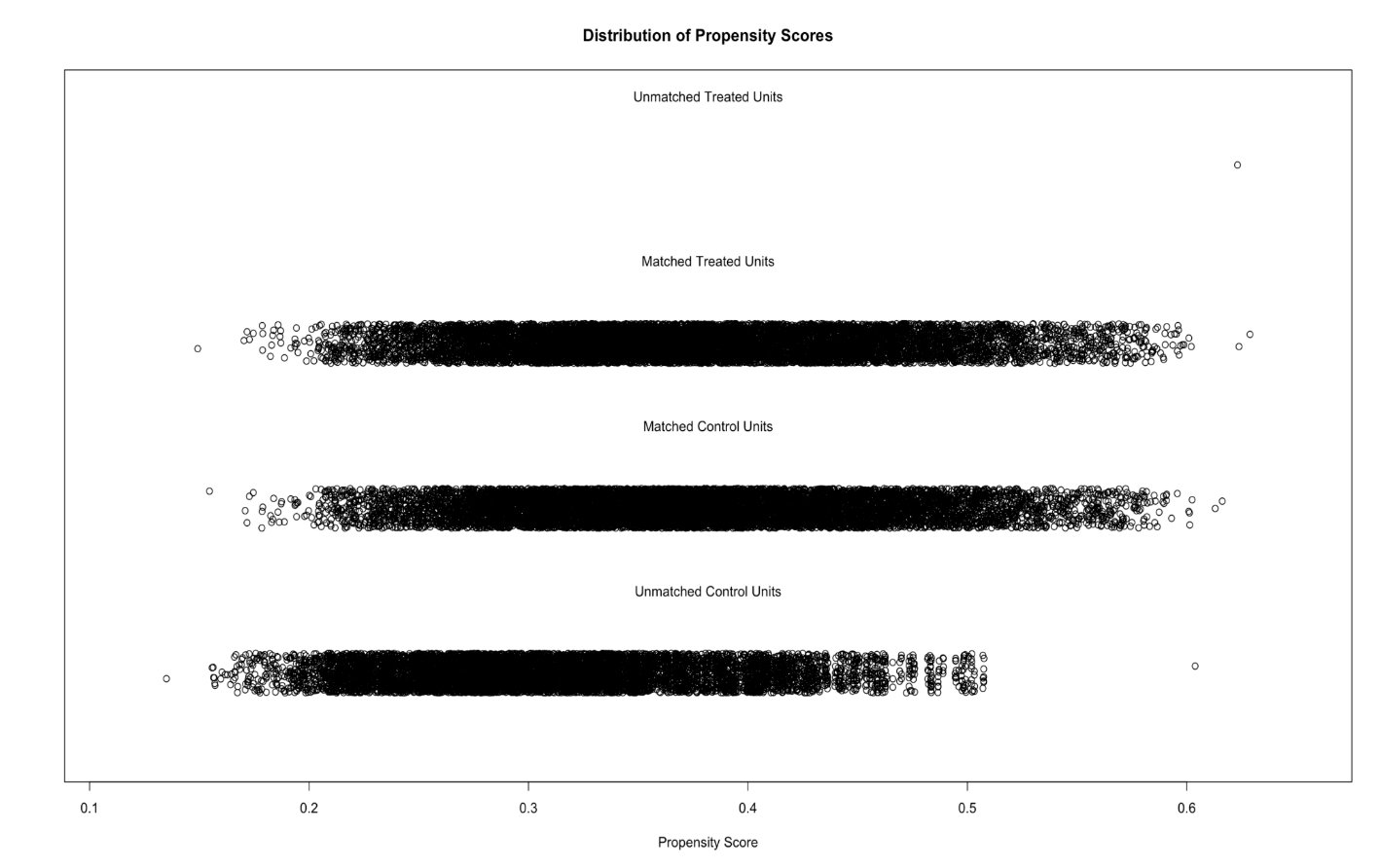

Supplement: Supplementary file 1 — Additional file 1: Figure S1. Covariate balance displayed via mean standardized differences before (white) and after propensity score-matching of the main analysis. Figure S2. Distribution of Propensity Scores before and after propensity score-matching in the main analysis. Figure S3. Distribution of age between groups (by period) in the main analysis after propensity score-matching. Figure S4. Distribution of the DECREASE III score between groups (by period) in the main analysis after propensity score-matching. Figure S5. Distribution of Length of Surgery between groups (by period) in the main analysis after propensity score-matching. Figure S6. Distribution of the ASA score between groups (by period) in the main analysis after propensity score-matching. Figure S7. Distribution of the Sex between groups (by period) in the main analysis after propensity score-matching. Figure S8. Distribution of the proportion of patients with cardiac surgery between groups (by period) in the main analysis after propensity score-matching. Figure S9. Covariate balance displayed via mean standardized differences before (white) and after propensity score-matching in the analysis of all patients receiving midazolam vs. no midazolam, irrespective of the admission date. Figure S10. Distribution of Propensity Scores before and after propensity score-matching in the in the analysis of all patients receiving midazolam vs. no midazolam, irrespective of the admission date. Table S1. Missing values in the various subsets of this analysis. Table S2. Baseline characteristics of included patients in period 1 (December 1, 2017 – November 31, 2018) and 2 (December 1, 2018 – November 31, 2019). Table S3. Baseline characteristics before and after propensity-score matching of patients receiving Midazolam vs. no Midazolam during the observation period of two years irrespective of admission date. Table S4. Outcome before and after propensity-score matching of patients receiving Midazolam vs. no Midazolam [file 13741_2025_568_MOESM1_ESM.docx]
